# A COI Nonsynonymous Mutation as Diagnostic Tool for Intraspecific Discrimination in the European Anchovy *Engraulis encrasicolus* (Linnaeus)

**DOI:** 10.1371/journal.pone.0143297

**Published:** 2015-11-24

**Authors:** Anna Maria Pappalardo, Concetta Federico, Giorgio Sabella, Salvatore Saccone, Venera Ferrito

**Affiliations:** Department of Biological, Geological and Environmental Sciences, Sec. Animal Biology “M. La Greca”, University of Catania, Catania, Italy; Kunming Institute of Zoology, Chinese Academy of Sciences, CHINA

## Abstract

The European anchovy, *Engraulis encrasicolus*, is currently one of the principal target species for commercial fisheries in Europe. In this study, the mitochondrial Control Region (*CR*) and the Cytochrome Oxidase I (*COI*) mitochondrial gene were analyzed in 74 specimens of *E*. *encrasicolus* from four localities in the central Mediterranean. In both populations, the two markers revealed the presence of two main haplogroups, A and B, already detected in previous investigations of different classes of molecular markers. Both *CR* and *COI* markers consistently identified two haplogroups. The *COI* sequence analysis identified a non-synonymous transversion (T to G) at position 116 of the translated sequence, resulting in an amino acid change. All *COI* sequences of haplogroup A had an amino acid sequence with alanine in this position, while serine was present in the same position in haplogroup B. The two haplogroups A and B were also discriminated by the variable number of TACA elements at the 5’-end of the mitochondrial *CR*. The selection tests applied to the *COI* dataset revealed that codon 116 was not under positive selection, that seven amino acid changes were under purifying selection, and that two amino acids were under episodic positive selection.

## Introduction

The European anchovy, *Engraulis encrasicolus* (Linnaeus, 1758), is a clupeoid pelagic species widely distributed in the Mediterranean, Black and Azov Seas, Eastern Atlantic coastline from Norway to Angola [1, 2], and around the tip of southern Africa [3, 4]. Its biology has received particular attention due to the commercial interest of its fisheries [5 — 8]. In the last twenty years a number of scientific studies have focused on the detection of genetic population structure in this commercially important species, through morphological and molecular analyses. Molecular studies described a complex genetic structure of *E*. *encrasicolus* using allozymes, mitochondrial DNA (mtDNA) and nuclear markers [9 –14]. In the Atlantic Ocean and the Mediterranean Basin, ten genetically differentiated European anchovy populations have been identified by surveys of the variability of the mitochondrial control region with implications for the management of fishery stocks [15, 16]. In particular, mtDNA, SNPs and nuclear intron markers have all supported the presence of two sympatric clades, named phylads or haplogroups A and B. The distribution of these haplogroups differs among natural populations but shows a constant proportion in each population, over time [15, 17–19]. Clade B prevails at the northern and southern high latitudes with frequencies decreasing towards the tropics, whereas clade A is present with higher frequencies in the tropical and subtropical areas [19]. Studies by Magoulas et al. [17, 18] and Grant [4] pointed out the role of climate change during Pleistocene glaciations, suggesting that the two phylads evolved in isolation in an Atlantic refugium and in the Mediterranean, respectively. It has also been suggested that the two mtDNA clades can also occur in simpatry, and that temperature seems to have shaped their relative frequency, as a result of range expansion and post-glacial secondary mixing [18, 19]. European anchovy population dynamics and distribution patterns are known to be strongly dependent on the environment. Thus, this species has been considered an ideal organism to study both the adaptative behaviour of small pelagic fish under different environmental conditions [19], and to understand the effect of quaternary climatic fluctuations on the distribution of marine organisms [14]. More specifically, studies by Silva et al. [14] demonstrated that the high mobile nature of anchovies allowed them to escape the adverse temperatures during the last glacial maximum without losing genetic diversity. Further, Silva et al. [19] detected positive selection in a single codon of the mitochondrial cytochrome b (cytb) gene in clade B, geographically correlated with environmental temperature. In the Mediterranean basin, within a latitudinal range of about 10° (45°-35°N) the annual average sea surface temperature goes from 15°C, in the northern Adriatic to 20°C in the Strait of Sicily or Sicilian Channel between the Sicilian and the North African coasts [20]. Moreover, the main water masses present in this channel, connecting the western and the eastern basins of the Mediterranean Sea, are characterized by a complex multi-scale thermohaline circulation, driven by ocean currents, wind effects, and mesoscale activity. All these environmental features are known to modify the temperature regime of the surface waters in the Sicilian anchovy habitat [21, 22]. Although the Sicilian Channel can be considered a key zone in the Mediterranean basin for the European anchovy population structure, few genetic studies [23] have been carried out in this area that was identified by Garcia Lafuente et al. [24] as one of the main spawning area of *E*. *encrasicolus*. Based on the considerations above, in this study the genetic structure of four populations of European anchovy from the Tyrrhenian, Ionian and Adriatic seas and the Sicilian Channel was investigated through the sequence analysis of two mitochondrial markers already used to detect A and B haplogroups in *E*.*encrasicolus* [16, 25], namely the noncoding Control Region (*CR*) and the coding Cytochrome Oxidase I (*COI*). The *CR* has been often used to infer intraspecific genetic structure and population demographic history [26–32]. Partial sequences of the mitochondrial Cytochrome c Oxidase I (*COI*) gene, a highly conserved, bioenergetic gene encoding for protein subunits of the respiratory chain [33], have been used to investigate the phylogeny of several animal taxa, including fishes, and as barcode sequences [34–39]. The aims are: i) to explore the nucleotide sequences of the two mitochondrial markers to unveil the molecular traits discriminating the two haplogroups of *E*. *encrasicolus*; ii) starting from the main conclusion of Silva et al. [19], and considering that a thermal cline is present between the Adriatic and the Sicilian Channel [20], to explore whether selection acted on the COI gene, using tests of recombination and selection based on different models of evolution.

## Materials and Methods

### Sampling, DNA extraction and PCR amplification

A total of 74 adult individuals of morphologically identified *E*. *encrasicolus* (deposited as vouchers at the Department of Biological, Geological and Environmental Science, Section of Animal Biology, in Catania, Italy) from four populations were collected in the central Mediterranean from 2012 to 2013 (Table 1).

**Table 1:** Diversity measures for the collecting sites of *Engraulis encrasicolus* for *CR* and *COI*: number of sequences (*n*), number of haplotypes (*N*_*h*_), haplotype diversity (*h*), nucleotide diversity *(π*) and relative standard deviation (SD).

				Control region			Cytochrome Oxidase I		
Location	Code	Date of collection	*n*	*N*_*h*_	*h* ± SD	*π* ± SD (10^−2^)	*N*_*h*_	*h* ± SD	*π* ± SD (10^−2^)
Sicilian Channel	SIC	October 2012	18	11	0.941 (0.033)	0. 025 (0.002)	12	0.948 (0.033)	0.012 (0.002)
Ionian Sea	ION	September 2013	20	13	0.947 (0.032)	0.029 (0.002)	10	0.837 (0.076)	0.012 (0.001)
Adriatic Sea	ADR	October 2013	18	11	0.908 (0.051)	0.023 (0.004)	11	0.817 (0.095)	0.008 (0.002)
Tyrrhenian Sea	TYR	March 2014	18	9	0.908 (0.039)	0.029 (0.003)	10	0.941 (0.029)	0.009 (0.001)
					overall 0.98 (0.005)			overall 0.93 (0.024)	

Fishes were obtained from artisanal fish landing sites, at night. Fish samples were collected off Porticello (38°05’05”N-13°32’47”E, Tyrrhenian Sea), Riposto (37°43’10”N-15°15’36”E, Ionian Sea), Porto Empedocle (37°14’59”N-13°31’02”E, Sicilian Channel) and Ancona (43°46’47”N-14°09’37”E, Adriatic Sea). Fishes were transferred to 95% ethanol before further processing in the laboratory. No experimentation with animals was performed. No other ethical issues applied to the present research. Total genomic DNA was extracted from muscle tissue (25–30 mg) using the DNeasy tissue kit (Qiagen, Hilden, Germany) following the manufacturer’s instructions. All PCR amplifications were carried out in 25 μl using approximately 50 ng of the isolated DNA as a template. In addition, each PCR reaction contained 16Taq DNA polymerase buffer (supplied by the respective Taq DNA polymerase manufacturer), 1.5–2 mM of MgCl2, 200 mM of each dNTP, 10 pmols of each primer and 0.5 U of Taq DNA polymerase (Platinum Taq DNA polymerase, Invitrogen). Amplification and sequencing of the mtDNA *COI* gene was performed using the primers FishF1 and FishR1 described in Ward et al. [38]. Thermal cycles involved an initial denaturing step of 2 min at 94°C, followed by 35 cycles of denaturation at 94°C for 30 s, annealing at 52°C for 45 s and extension at 72°C for 1 min. Negative controls were included in all PCR runs to control for cross-contamination. Double-stranded products were checked by agarose gel electrophoresis, purified with the Qiaquick PCR purification kit (Qiagen) and subsequently sequenced in the forward and reverse directions. For PCR amplification of mitochondrial *CR*, two primers (ENG_FW: 5’- TGTAAAACGACGGCCAGTTTCTAAAGTTAAACTACCCTCT; ENG_REV1: 5’-CAGGAAACAGCTATGACTTAAGTGAACGCTCGGCATGG-3’) specific to European anchovy were designed from the species’ complete mtDNA sequence.

The PCR conditions were as follows: initial denaturation at 94°C for 5 min, followed by denaturation at 94°C for 45 sec, annealing at 55°C (45 sec) and extension at 72°C (1 min) repeated for 35 cycles and with a final extension step at 72°C for 5 min. Negative controls were included in all PCR runs to control for cross-contamination. Double-stranded products were checked by agarose gel electrophoresis and purified with the Qiaquick PCR purification kit (Qiagen). PCR products were subsequently sequenced in the forward and reverse direction by Genechron (http://www.genechron.it/index.php/sanger-sequencing) using an ABI Prism 3100 automated sequencer (Applied Biosystems) and M13 forward and M13 reverse primers for sequencing.

### Data analysis

The chromatograms obtained were assembled and checked by eye. Edited sequences were aligned using the default settings in ClustalX 2.0 software [40] and the alignment was manually revised in BioEdit (http://www.mbio.ncsu.edu/bioedit/bioedit.html). The number of polymorphic sites and genetic diversity indexes (number of haplotypes, haplotype diversity and nucleotide diversity) were calculated using DNAsp 5.1 [41] (Table 1). An analysis of molecular variance (AMOVA), implemented in ARLEQUIN version 3.5.2.1 [42] was applied to the distance matrix to estimate variance components and Φ*st* values. A haplotype network was built using the median joining (MJ) algorithm [43] in the program Network 4.6.1.3. (Fluxus-engineering.com). Nucleotide diagnostics (NDs) [44, 45] for each European anchovy haplogroup were detected after the editing of sequence alignments using CLC Sequence Viewer 7.6 (www.clcbio.com). To correctly assign each sequence to haplogroup A or B, we used the mtDNA *CR* sequences of the population in the Canary Islands where 100% of the individuals belong to clade A, as a reference (GenBank accession numbers: HQ215641, JQ595031, JQ595040, JQ595059, JQ595109, JQ595102, JQ595187-JQ595216) [16]. After alignment, the mtDNA *CR* sequences that clustered with those of the Canary Islands were considered to belong to clade A.

### Tests of recombination and selection

To test the presence of mitochondrial *COI* recombinants, we used the Genetic Algorithms for Recombination Detection (GARD) [46] (HyPhy package, accessed at www.datamonkey.org). To test the presence of selection on *COI*, the Z-test [47] was performed in MEGA v.6 [48]. To determine the site-specific selection pressures acting on the *COI* gene, the single-likelihood ancestor counting (SLAC), fixed-effects likelihood (FEL), internal FEL (IFEL), fast unconstrained Bayesian approximation (FUBAR) and mixed effects model of evolution (MEME) methods were applied. Sites with *p*-values less than 0.05 for SLAC, FEL, IFEL and MEME, as well as sites with posterior probability of more than 0.9 for FUBAR were all considered as being under selection [19]. To visualize the structural position of positively selected sites, the secondary structure of the barcode region was predicted using the PSIPRED method [49, 50]. The 3-dimensional structure was drawn with Jmol (accessed at http://www.jmol.org).

The 217 *COI* amino acid sequence examined in this study is homologous to the 18–234 fragment of the full bovine *COI* protein sequence (http://c.expasy.org/uniprot/P00396#seq).

## Results

### Mitochondrial *CR* analysis and NDs

**Fig 1:**
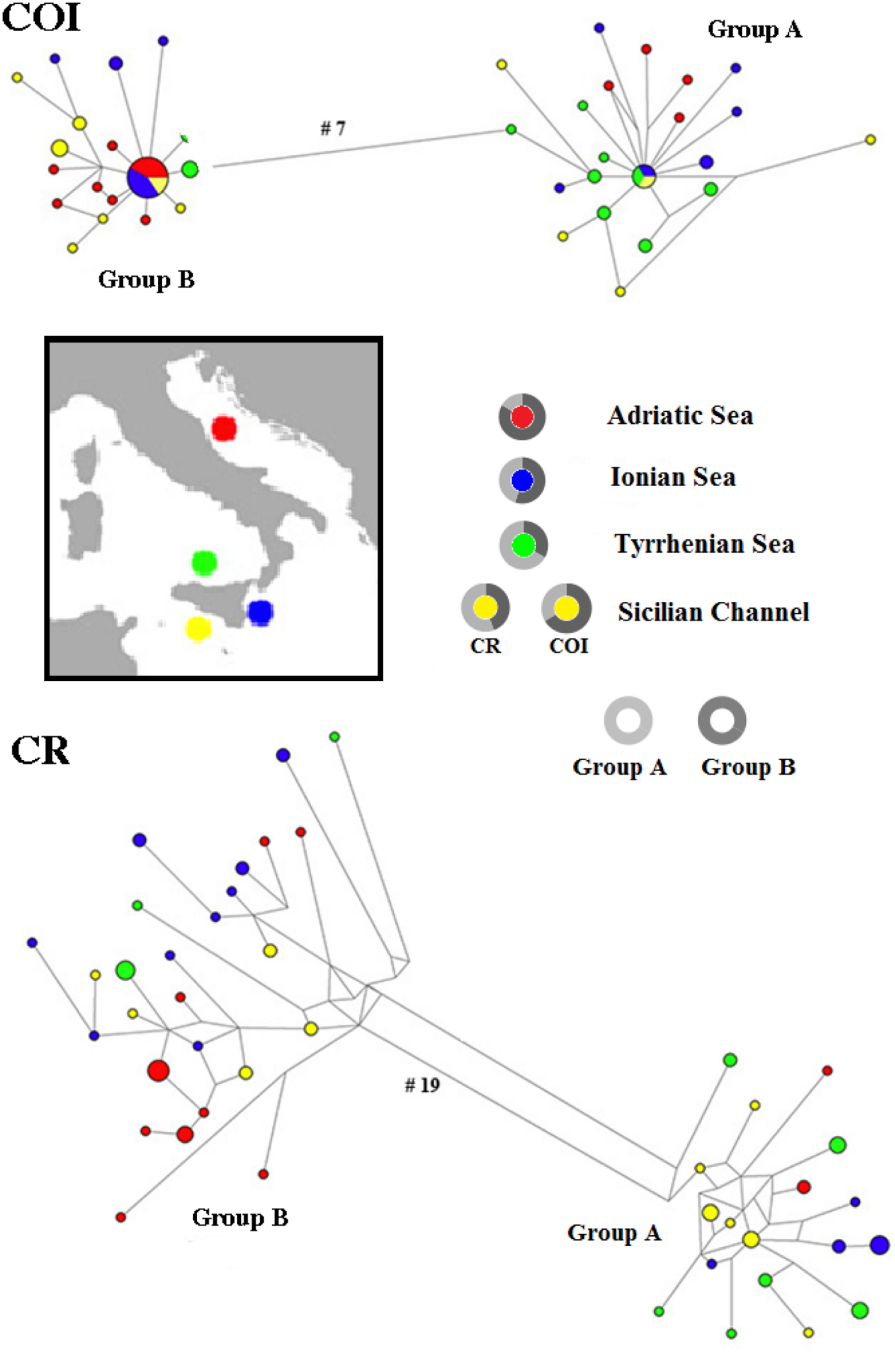
Median-joining network for the COI and CR of *Engraulis encrasicolus*. The area of the circles is proportional to each haplotype frequency. Colors refer to the region in which haplotypes were found. In the case where haplotypes are shared among regions, pie charts show the relative haplotype frequency in each region. Grey circles represent mtDNA CR and COI clades A (light grey) and B (dark grey) proportions. Only in the Sicilian Channel the relative proportion of the two clades is different for the two markers.

For the *CR*, after alignment, a 577 bp fragment was analyzed. We obtained 74 sequences defining 44 haplotypes (S1 File), with a total of 76 polymorphic sites including 18 singletons and 58 parsimoniously informative sites. No shared haplotypes were found among populations. High values of haplotype and nucleotide diversity indices were found in Ionian Sea and Sicilian Channel populations (Table 1). AMOVA revealed overall low but statistically significant genetic structuring of the analyzed samples (Φst = 0.11 p< 0.0001 +/- 0.0000). The majority of the variance was distributed within (89.50%) and not among (10.50%) samples (S2 File). Overall haplotype diversity of mitochondrial *CR* (0.98) was much higher than *COI* (Table 1). The MJ network showed the clear splitting of the control region haplotypes into two groups including samples from all populations (Fig 1).

The inclusion of mtDNA *CR* sequences of the Canary Islands [16] in the dataset (not shown) allowed the identification of haplogroup A. Notably, four or five 5’-TACA-3’ repeats were found at the 5’ end of the *CR*, at positions 82, 120, 124, 140 and 160. More specifically, no TACA repeats at position 124 were found in haplotypes of haplogroup A, while 65% of haplotypes in haplogroup B had TACA repeats in this position. The TACA at position 140 was not found in 96% of the haplotype sequences in haplogroup B, while all haplotypes in haplogroup A had TACA repeats in this position. A total of four NDs, including three transversions (positions: 37, 222 and 467) and one transition at position 333, discriminated haplogroups A and B (S2 File).

### *COI* sequence analysis, NDs

Unambiguously aligned sequences were obtained for 651 bp of *COI* sequence from 74 tissue samples of *E*. *encrasicolus*. All sequences were deposited in GenBank under the accession numbers reported in S1 File. No insertions, deletions or stop codons were observed. The lack of stop codons is consistent with all amplified sequences being functional mitochondrial *COI* sequences, along with the fact that all amplified sequences were of the same length (651 bp). This suggests that NUMTs (nuclear DNA sequences originating from mitochondrial DNA sequences) were not sequenced (vertebrate NUMTS are generally smaller than 600 bp) [51]. A total of 57 variable nucleotide sites with 28 parsimony-informative sites defined 39 distinct anchovy haplotypes (S1 File). Two haplotypes were found in different sampled regions: H5 in the Adriatic Sea, Sicilian Channel and Ionian Sea; and H18 in the Tyrrhenian Sea, Sicilian Channel and Ionian Sea. High values of haplotype diversity were found in the Sicilian Channel and Tyrrhenian Sea samples (Table 1). AMOVA revealed overall low but statistically significant genetic structuring of the analyzed samples (Φst = 0.11 p< 0.007+/- 0.0023). The majority of the variance was distributed within (88.88%) and not among (11.12%) samples (S3 File). The MJ network showed the clear splitting of the *COI* haplotypes into two groups (A and B) including samples from all populations (Fig 1).

**Fig 2:**
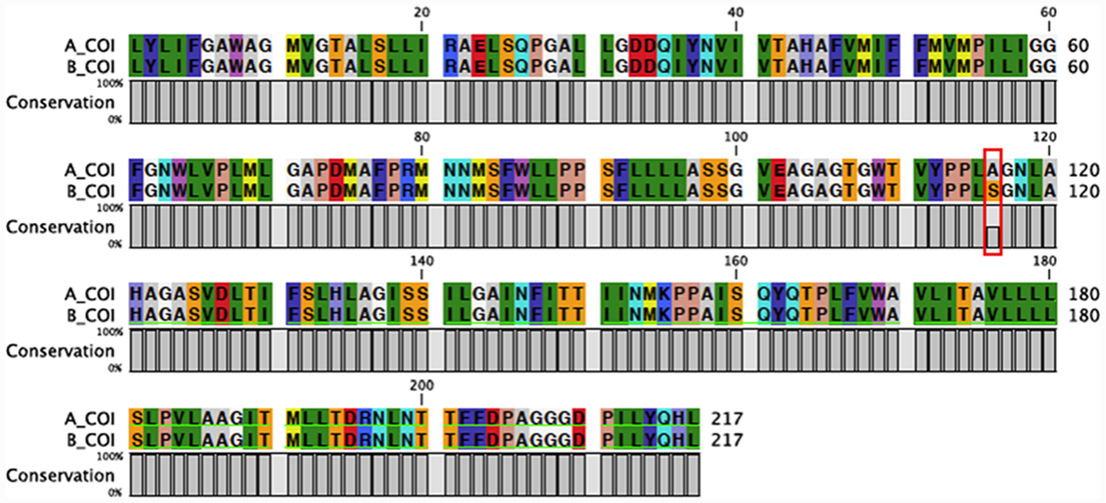
*COI* amino acid aligned sequences of *Engraulis encrasicolus*. The red box indicates the amino acid change. Serine (S) is found in haplogroup B and alanine (A) in haplogroup A.

A total of six nucleotide diagnostics (NDs) for haplogroup discrimination of *E*. *encrasicolus* (group A and group B) are identified in the S4 File. Four of these are transitions (positions: 344, 358, 421 and 454) and two are transversions (positions: 347 and 367). All substitutions are silent with the exception of the G to T transversion at position 347, which changes the amino acid from alanine (A) to serine (S) at position 116 in 58% of the sequences (Fig 2).

All amino acid sequences of haplogroup A have an alanine at position 116, and the serine is found in the same position in all sequences of haplogroup B (S5 File).

### Tests of recombination and selection

No evidence for recombination was found in the *COI* dataset with GARD. The Z-test rejected the null hypothesis of strict neutrality (d_NON-SYNONYMOUS_ = d_SYNONYMOUS_; p = 0,000) in favor of the alternative hypothesis of positive selection (d_N_ > d_S_) in clade B. Seven amino acids were under purifying selection and two under positive selection (codons 11 and 121). Episodic positive selection was detected with the MEME method for codon 11 and 121 in clade B (Table 2) and with MEME and IFEL methods for codon 121 in clade A.

**Table 2:** Positively and negatively selected sites in the cytochrome oxidase I gene estimated by FUBAR, SLAC, IFEL, FEL and MEME models.

Codon		11	42	82	104	113	121	150	155	211
Selection type		positive	purifying	purifying	purifying	purifying	positive	purifying	purifying	purifying
all	FUBAR		##		#	#		##		##
	SLAC									*
	IFEL									
	FEL		*	*		*		*	*	*
	MEME	***					**			
Clade A	FUBAR		##			##				##
	SLAC									
	IFEL						*			
	FEL					*				*
	MEME						**			
Clade B	FUBAR		##		#	#		##		##
	SLAC									
	IFEL									
	FEL		*	*		*		*	*	*
	MEME	***					**			

^#^bpp ≥0.9;

^##^bpp ≥0.95;

^###^bpp ≥0.99

*p < 0.05;

** p <0.01;

***p <0.001

**Fig 3:**
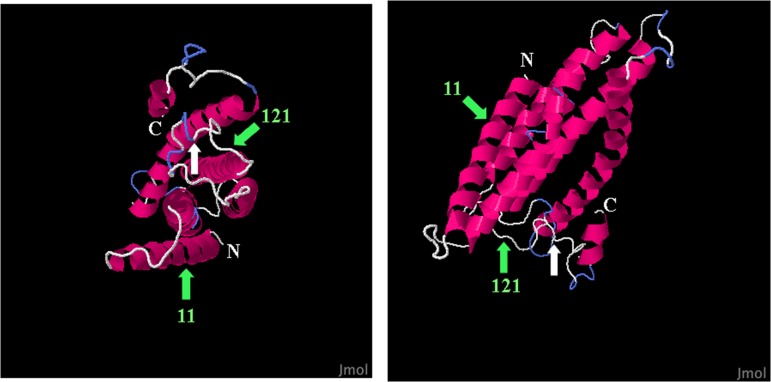
3-dimensional structure of the *COI* barcode region and structural location of positively selected sites (green arrows). On the right, lateral view with the amino terminal tail (N) facing towards the mitochondrial matrix surface and the carboxy terminal tail (C) facing towards the inter-membrane space surface. On the left, a top view. White arrow point to the neutral non-synonymous change at position 116 allowing for haplogroup discrimination.

In haplogroup A histidine replaced methionine at codon 11 and proline replaced histidine at codon 121. The amino acids in the “Folmer region” (the standard barcode region) form five helices (http://c.expasy.org/uniprot/P00396#seq). Based on the prediction of the protein secondary structure, codon 116 is located in the third coil toward the inter-membrane space. Codons 11 and 121 under episodic positive selection are located in the first helix and in the third coil, respectively (Fig 3).

### Comparison between *CR* and *COI* results

The two divergent haplogroups detected with both mitochondrial markers were separated by 7 point mutations in the *COI* network and by 19 point mutations in the *CR* network. The percentage of divergence between the two clades was 4.4% for *CR* and 2% for *COI*. Substantial agreement exists between *CR* and *COI* in the assignment of each individual to the same haplogroup for all populations except for Sicilian Channel (Fig 1). Overall, 46% of the *CR* haplotypes and 42% of the *COI* haplotypes were included in clade A. More specifically, clade B prevails in the Adriatic and in the Ionian samples with both markers.

## Discussion

Both mitochondrial molecular markers identified two main haplogroups which were identified as the haplogroups A and B, by comparison with some mtDNA CR sequences from the Canary Islands [16]. Both revealed low but significant genetic structuring with Φst values congruent with those detected in other studies [18]. The average *COI* haplotype diversity (*h* = 0.93) is lower than for *CR* (*h* = 0.98). These results indicate the effectiveness of *COI* sequence variation in detecting genetic structure in *E*. *encrasicolus* and other teleosts such as swordfish and lanternfish species [52, 53]. The distribution of TACA repeats in the *CR* allowed to discriminate the two haplogroups of European anchovy; a similar result was obtained in *Xiphias gladius* where TACA repeats discriminated geographical stocks of this species [52]. A relevant result was the detection of a non-synonymous substitution at the site 347 of the *COI* sequence. It should be noted that the substitution involved an aliphatic and apolar amino acid (alanine) and a hydroxylated and polar amino acid (serine), which are functionally different. This is contrary to the evidence that substitutions in the *COI* amino acid sequence are known to be frequent among physicochemically similar amino acids [54]. The relevance of this finding should be assessed given that the 655 bp region of *COI* codes for 218 amino acids and tends to be strongly conserved due to functional constraints on amino acid substitutions. Studies by Ward and Holmes [54] on the nucleotide and amino acid variability of *COI* in fishes demonstrated that the ratio of non-synonymous to synonymous substitutions is much lower than one, indicating that this gene is subject to strong purifying selection. In exploring the role of the mutation and selection in mitochondrial protein-coding genes, Castellana et al. [33] noted the strong influence of purifying selection on all mitochondrial genes and especially on *COI*, *COII*, *COIII* and *Cytb*, with crucial functions in the respiratory chain and thus strongly preserved. However, the frequently discussed role of positive selection in thermal adaptation and aerobic performance has also been demonstrated for these genes in invertebrates and vertebrates [55–57], making the implicit assumption of neutrality of mitochondrial markers no longer valid [58]. The European anchovy has been studied in this respect by Silva et al. [19], which detected positive selection in a single codon of the mitochondrial *Cytb* gene in the clade B strongly correlated with the temperature. The authors discuss the functional role of the non-synonymous substitution that they identified, suggesting that it may have an impact on the overall metabolic performance of clade B. The tests of selection applied to our *COI* dataset indicated positive selection only for clade B through the Z test. Seven codons were found to be under purifying selection and two (codons 11 and 121) under episodic positive selection. However, codon 116, located near codon 121 on the third coil, was not among the codons under selection. Codons under episodic positive selection experience purifying selection for the majority of their evolution, interspersed with bursts of positive selection that may occur only in restricted lineages [59]. Mutations at such sites may experience transient positive selection, followed by purifying selection to maintain the change, and could play a key role in adaptive evolution [59, 60]. Molecular footprints of episodic positive selection have been underestimated until now and the MEME method shows a great power to unveil sites under this model of selection. Tomasco & Lessa [57] found two sites in both *COII* and *Cytb*, using MEME, showing footprints of episodic positive selection, likely functionally relevant.

We have not evaluated the functional implications of the amino acid substitutions found in the *COI* gene, all involving residues with dissimilar properties. However, amino acid substitutions not involved regions known to have highly conserved, important functions (e.g. d-pathway or proton pathway or binding the heme or cytochrome c interactions) [54]. We could only argue that subtle modifications may alter interactions between proteins and their stability, modifying the performance of oxidative phosphorylation processes under different environmental selective pressures [19, 56, 61]. The above results are consistent with the main conclusions of previous investigations aimed to demonstrate the high responsiveness of European anchovy to the changing environment [14, 19] and the strong influence of contemporary and historical processes in shaping the distribution and the genetic structure of this small pelagic species [18].

Our sequence analyses revealed the molecular traits of the mitochondrial *CR* and *COI* fragments useful to discriminate haplogroups of *E*. *encrasicolus*. The application of the MEME method unveiled that two sites of *COI* were under episodic positive selection. These results add to the emerging data from the most recent surveys on adaptive selection of mitochondrial DNA, suggesting that episodic positive selection affects a large proportion of sites, also in a highly conserved gene such as *COI*.

## Supporting Information

S1 File List of *CR* and *COI* haplotypes from *E*. *encrasicolus* samples. (XLSX)

S2 File Variable nucleotide sites in the 575–577 bp sequences of the European anchovy *CR* examinated in this work. Marked haplotypes belong to haplogroup A.
(PDF)

S3 File Analysis of Molecular Variance (AMOVA) calculated for the populations examined in this work with Arlequin ver 3.5.2.1. (XLSX)

S4 File Variable nucleotide sites in the 651 bp sequences of the European anchovy *COI* gene examinated in this work. Marked haplotypes belong to haplogroup A. The non-synonymous mutation highlighted in red is diagnostic for discrimination of A and B haplogroups.
(PDF)

S5 File Variable codon sites in the 217 amino acids of the European anchovy *COI* gene examinated in this work. The codon 116 highlighted in red is diagnostic for discrimination of A and B haplogroups. The codons 11 and 121 highlighted in grey are under episodic positive selection.
(PDF)
