# Supplementary material for: A COI Nonsynonymous Mutation as Diagnostic Tool for Intraspecific Discrimination in the European Anchovy Engraulis encrasicolus (Linnaeus)
Source: PLoS One. 2015 Nov 24;10(11):e0143297. doi: 10.1371/journal.pone.0143297 (PMC4657973; doi:10.1371/journal.pone.0143297)

```
                                                     20                            40                            60                            80
HAP11_DLOOP ☒  AAATAGTGCT TGATGCCCTT AGACAGTTCA AGCACTCGTT CATGACTGCG CAGAG-ATTG ATGGACATAT ATGTATTATT 79
HAP39_DLOOP ☒  .......... .......... ..G....... ......T... .......... .....C...C .......... .......... 80
HAP38_DLOOP ☒  .......... .......... ..G....... .......... .......... .....C...C .......... .......... 80
HAP25_DLOOP ☒  ..G....... .......... ..G....... .......... .......... .....C...C .......... .......... 80
HAP28_DLOOP ☒  ..G.G..... .......... ..G....... .......... .......... .....C...C .......... .......... 80
HAP24_DLOOP ☒  .......... .......... ..G....... .......... .......... .....C...C .......... .......... 80
HAP30_DLOOP ☒  ..G....... .......... ..G....... .......... .......... .....C...C .......... .........C 80
HAP34_DLOOP ☒  .......... .......... ..G....... .......... .......... .....C...C .......... .......... 80
HAP35_DLOOP ☒  .......... .......... ..G....... .......... .......... .....C...C .......... .......... 80
HAP23_DLOOP ☒  .......... .......... ..G....... ......T... .......... .....C...C .......... .......... 80
HAP29_DLOOP ☒  ..G....... .......... ..G....-.. .......... .......... .....-...- .......... .......... 77
HAP10_DLOOP ☒  .......... .......... ..G....... .......... .......... .....C...C .......... .......... 80
HAP27_DLOOP ☒  ..G....... .......... ..G....... .......... .......... .....C...C .......... .......... 80
HAP37_DLOOP ☒  ..G....... .......... ..G....... ......T... .......... .....C...C .......... .......... 80
HAP26_DLOOP ☒  ..G....... .......... ..G....... .......... .......... .....C...C .......... .......... 80
HAP36_DLOOP ☒  .......... .......... ..G....... .......... .......... .....C...C .......... .......... 80
HAP21_DLOOP ☒  ...C...... .......... ..G....... ......T... .......... .....C...C .......... .......... 80
HAP22_DLOOP ☒  ..GC...... .......... ..G....... ......T... .......... .....C...C .......... .......... 80
HAP12_DLOOP ☐  ..G....... .......... ..G....... ......G... .......... .G...C...C .......... .......... 80
 HAP9_DLOOP ☐  ..G....... .......... ..G....... ......G... .......... .G...C...C .......... .......... 80
HAP44_DLOOP ☐  ..G....... .......... ..G....... ......G... .......... .G...C...C .......... .......... 80
HAP15_DLOOP ☐  ..G....... .......... ..G....... ......G... .......... .G...C...C .......... .......... 80
HAP14_DLOOP ☐  ..G....... .......... ..G....... ......G... .......... .G...C...C .......... .......... 80
HAP13_DLOOP ☐  ..G....... .......... ..G....... ......G... .......... .G...C...C .......... .......... 80
HAP16_DLOOP ☐  ..G....... .......... ..G.....T. ......G... ...-...... .....C...C .......... .....G.... 79
 HAP6_DLOOP ☐  ..G....... .........A GC........ ......G... .......... .....-...T .......... .......... 79
 HAP5_DLOOP ☐  ..G....... .......T.A G-........ ......G... .......... .....-...C .......... .......... 78
 HAP7_DLOOP ☐  ..G....... ....A..... ....G..-.. ......A... .......... .....-...C .......... .......... 78
HAP33_DLOOP ☐  ..G....... .......... ..G....... ......A... .......... .....C...C .......... .........C 80
HAP42_DLOOP ☐  ..G....... .......... ..G....... ......G... .......... .....C...C .......... .......... 80
HAP32_DLOOP ☐  ..G....... .......... ..G....... ......G... .......... .....C...C .......... ......-G.. 79
HAP43_DLOOP ☐  ..G....... .......... ..G....... ......G... .......... .....C...C .......... .......... 80
HAP19_DLOOP ☐  ..G....... .......... ..G....... ......G... .......... .....C.G.C .......... .......... 80
HAP18_DLOOP ☐  ..G....... .......... ..G....... ......G... .......... .....C...C .......... .......... 80
 HAP8_DLOOP ☐  ..G....... .......... ..G....... ......G... .......... .....C...C .......... .......... 80
HAP40_DLOOP ☐  ..G....... .......... ..G....... ......G... .......... .....C...C .......... .......... 80
HAP31_DLOOP ☐  ..G....... .......... ..G....... ......G... .......... .....C...C .......... ........C. 80
HAP41_DLOOP ☐  ..G....... .......... ..G....... ......G... .......... .....C...C .......... .......... 80
HAP17_DLOOP ☐  ..G....... .......... ..G....... ......G... .......... .....C...C .......... .......... 80
HAP20_DLOOP ☐  ..G....... .......... ..G....... ......G... .......... .....C...C .......... .......... 80
 HAP1_DLOOP ☐  ..G....... .......... ..G....... ......G... .......... .....C...C .......... .......... 80
 HAP2_DLOOP ☐  .......... .......... ..G....... ......G... .......... .....C...C .......... .......... 80
 HAP3_DLOOP ☐  .......... .......... ..G....... ......G... .......... .....C...C .......... .......... 80
 HAP4_DLOOP ☐  ..G....... .......... ..G....... ......G... .......... .....C...C .......... .......... 80
  Consensus    AAGTAGTGCT TGATGCCCTT AGGCAGTTCA AGCACTGGTT CATGACTGCG CAGAGCATTC ATGGACATAT ATGTATTATT
```

Conservation

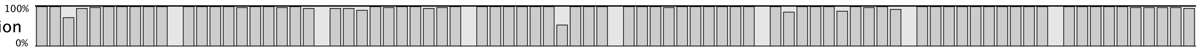

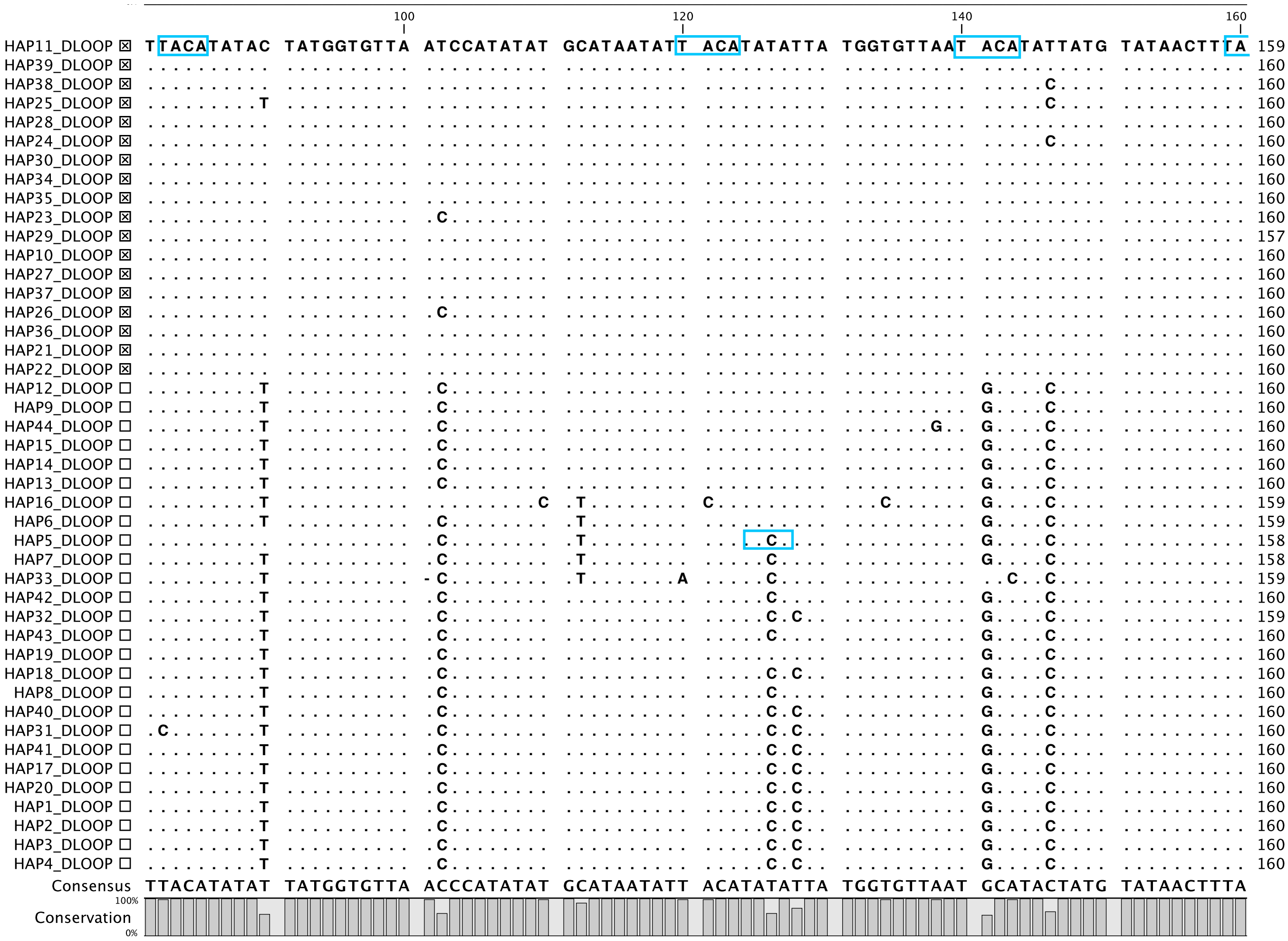
100
120
140
160
HAP11_DLOOP TTACATATAC TATGGTGTTA ATCCATATAT GCATAATATT ACATATATTA TGGTGTTAAT ACATATTATG TATAACTTTA 159
Consensus TTACATATAT TATGGTGTTA ACCCATATAT GCATAATATT ACATATATTA TGGTGTTAAT GCATACTATG TATAACTTTA
Conservation
100%
0%

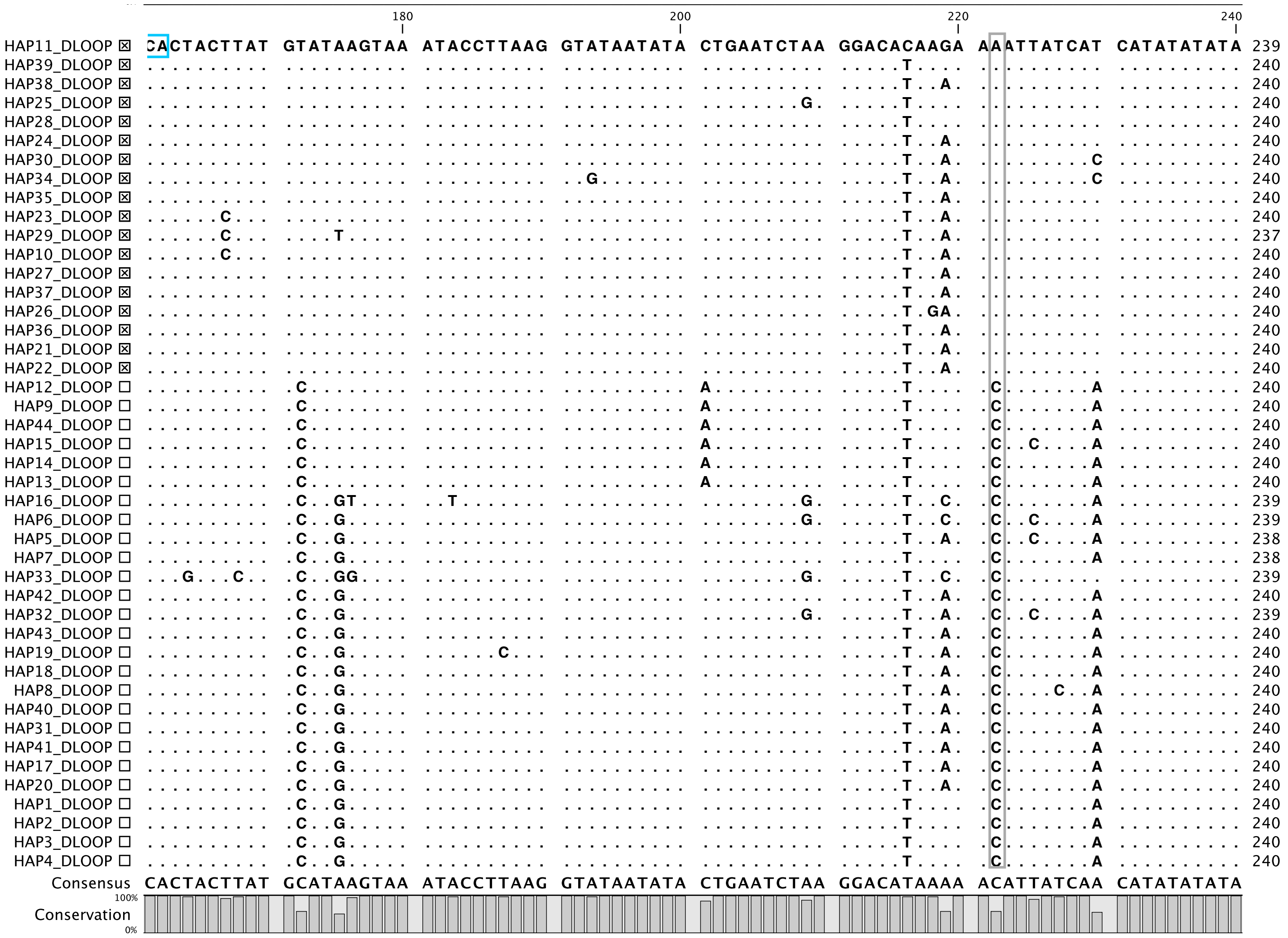
180 200 220 240
HAP11_DLOOP ⊠ CACTACTTAT GTATAAGTAA ATACCTTAAG GTATAATATA CTGAATCTAA GGACACAAGA AAATTATCAT CATATATATA 239
HAP39_DLOOP ⊠ ....T.... 240
HAP38_DLOOP ⊠ ....T..A. 240
HAP25_DLOOP ⊠ ....G. .....T.... 240
HAP28_DLOOP ⊠ ....T.... 240
HAP24_DLOOP ⊠ ....T..A. 240
HAP30_DLOOP ⊠ ....T..A. ....C 240
HAP34_DLOOP ⊠ ....G....... ....T..A. ....C 240
HAP35_DLOOP ⊠ ....T..A. 240
HAP23_DLOOP ⊠ ......C... ....T..A. 240
HAP29_DLOOP ⊠ ......C... ....T..... ....T..A. 237
HAP10_DLOOP ⊠ ......C... ....T..A. 240
HAP27_DLOOP ⊠ ....T..A. 240
HAP37_DLOOP ⊠ ....T..A. 240
HAP26_DLOOP ⊠ ....T.GA. 240
HAP36_DLOOP ⊠ ....T..A. 240
HAP21_DLOOP ⊠ ....T..A. 240
HAP22_DLOOP ⊠ ....T..A. 240
HAP12_DLOOP ☐ .... .C........ .... A......... .....T.... .C.......A 240
HAP9_DLOOP ☐ .... .C........ .... A......... .....T.... .C.......A 240
HAP44_DLOOP ☐ .... .C........ .... A......... .....T.... .C.......A 240
HAP15_DLOOP ☐ .... .C........ .... A......... .....T.... .C..C....A 240
HAP14_DLOOP ☐ .... .C........ .... A......... .....T.... .C.......A 240
HAP13_DLOOP ☐ .... .C........ .... A......... .....T.... .C.......A 240
HAP16_DLOOP ☐ .... .C..GT.... ..T....... ....G. .....T..C. .C.......A 239
HAP6_DLOOP ☐ .... .C..G..... ....G. .....T..C. .C..C....A 239
HAP5_DLOOP ☐ .... .C..G..... ....T..A. .C..C....A 238
HAP7_DLOOP ☐ .... .C..G..... ....T.... .C.......A 238
HAP33_DLOOP ☐ ...G...C.. .C..GG.... ....G. .....T..C. .C........ 239
HAP42_DLOOP ☐ .... .C..G..... ....T..A. .C.......A 240
HAP32_DLOOP ☐ .... .C..G..... ....G. .....T..A. .C..C....A 239
HAP43_DLOOP ☐ .... .C..G..... ....T..A. .C.......A 240
HAP19_DLOOP ☐ .... .C..G..... ......C... ....T..A. .C.......A 240
HAP18_DLOOP ☐ .... .C..G..... ....T..A. .C.......A 240
HAP8_DLOOP ☐ .... .C..G..... ....T..A. .C....C..A 240
HAP40_DLOOP ☐ .... .C..G..... ....T..A. .C.......A 240
HAP31_DLOOP ☐ .... .C..G..... ....T..A. .C.......A 240
HAP41_DLOOP ☐ .... .C..G..... ....T..A. .C.......A 240
HAP17_DLOOP ☐ .... .C..G..... ....T..A. .C.......A 240
HAP20_DLOOP ☐ .... .C..G..... ....T..A. .C.......A 240
HAP1_DLOOP ☐ .... .C..G..... ....T.... .C.......A 240
HAP2_DLOOP ☐ .... .C..G..... ....T.... .C.......A 240
HAP3_DLOOP ☐ .... .C..G..... ....T.... .C.......A 240
HAP4_DLOOP ☐ .... .C..G..... ....T.... .C.......A 240
Consensus CACTACTTAT GCATAAGTAA ATACCTTAAG GTATAATATA CTGAATCTAA GGACATAAAA ACATTATCAA CATATATATA
Conservation 100% 0%

```
                 260         280         300         320
                   |           |           |           |
HAP11_DLOOP ☒  ATCTAACCCA ACCAAGTAAC AATAA-AACT TAGACAGACA TAAACTGCAA ACAGAATACT CACAAAGAAC TCCAACACAG 318
HAP39_DLOOP ☒  .......... .......... .....-.... .......... .......... .......... .......... .......... 319
HAP38_DLOOP ☒  .......... .......... .....-.... .......... .......... .......... .......... .......... 319
HAP25_DLOOP ☒  .......... .......... .....-.... .......... .......... .......... .......... .......... 319
HAP28_DLOOP ☒  .......... .T........ .....-.... .......... .......... .......... .......... ......G... 319
HAP24_DLOOP ☒  .......... .T........ .....-.... .......... .......... .......... .......... ......G... 319
HAP30_DLOOP ☒  ..T....... .......... .....-.... .......... .......... .......... ...G...... ......G... 319
HAP34_DLOOP ☒  ..T....... .......... .....-.... .......... .......... .......... ...G...... ......G... 319
HAP35_DLOOP ☒  ..T....... .......... .....-.... .......... .......... .......... .......... ......G... 319
HAP23_DLOOP ☒  .......... .......... .....-.... .......... .......... .......... .......... ......G... 319
HAP29_DLOOP ☒  .......... .......... .....-.... .......... .......... .......... .......... .......... 316
HAP10_DLOOP ☒  .......... .......... .....-.... .......... .......... .......... .......... .......... 319
HAP27_DLOOP ☒  .......... .......... .....-.... .......... .......... .......... .......... ......G... 319
HAP37_DLOOP ☒  .......... .......... .....-.... .......... .......... .......... .......... ......G... 319
HAP26_DLOOP ☒  .......... .......... .....-.... .......... .......... .......... .......... ......G... 319
HAP36_DLOOP ☒  .......... .......... .....-.... .......... .......... .......... .......... ......G... 319
HAP21_DLOOP ☒  .......... .......... .....-.... .......... .......... .......... ...G...... ......G... 319
HAP22_DLOOP ☒  .......... .......... .....-.... .......... .......... .......... ...G...... ......G... 319
HAP12_DLOOP ☐  ..TA...... .T........ .....-.... .......... .......... .......... .......... .......... 319
 HAP9_DLOOP ☐  ..TA...... .T........ .....-.... .......... .......... .......... .......... .......... 319
HAP44_DLOOP ☐  ..TA...... .T........ .....-.... .......... .......... .......... .......... .......... 319
HAP15_DLOOP ☐  ..TA...... .T........ .....-.... .......... .......... .......... .......... .......... 319
HAP14_DLOOP ☐  ..TA...... .T.......G .....-.... .......... .......... .......... .......... .......... 319
HAP13_DLOOP ☐  ..TA...... .T.......G .....-.... .......... .......... .......... .......... ......G... 319
HAP16_DLOOP ☐  ..TA...... .......... .....-.... .......... .......... .......... .......... .......... 318
 HAP6_DLOOP ☐  ..TA...... .T........ .....-.... .......... .......... .......... ...G...... .......... 318
 HAP5_DLOOP ☐  ..TA...... .......... .....-.... .......... .......... .......... .......... .......... 317
 HAP7_DLOOP ☐  ..TA...... .T........ .....-.... .......... .......... .......... .......... .......... 317
HAP33_DLOOP ☐  ..TA...... .......... .....-.... .......... .......... .......... .......... ......G... 318
HAP42_DLOOP ☐  .CTA...... .T........ .....-.... .......... .......... .......... .......... .......... 319
HAP32_DLOOP ☐  .CTA...... .T.......T .....-.... .......... .......... .......... .......... .......... 318
HAP43_DLOOP ☐  .CTA...... .......... .....-.... .......... .......... .......... .......... .......... 319
HAP19_DLOOP ☐  .CTA...... .......... .....-.... .......... .......... .......... .......... .......... 319
HAP18_DLOOP ☐  .CTA...... .......... .....-.... .......... .......... .......... .......... .......... 319
 HAP8_DLOOP ☐  .CTA...... .......... .....-.... .......... .......... .......... .......... ......G... 319
HAP40_DLOOP ☐  .CTA...... .......... .....-.... .......... .......... .......... ...G...... ......G... 319
HAP31_DLOOP ☐  .CTA...... .......... .....-.... .......... .......... .......... .......... ......G... 319
HAP41_DLOOP ☐  .CTA...... .T........ .....-.... .......... .......... .......... .......... ......G... 319
HAP17_DLOOP ☐  .CTA...... .T........ .....-.... .......... .......... .......... .......... ......G... 319
HAP20_DLOOP ☐  .CTA...... .T........ .....-.... .......... .......... .......... .......... ......G... 319
 HAP1_DLOOP ☐  .CTA...... .......... .....-.... .......... .......... .......... .......... ......G... 319
 HAP2_DLOOP ☐  .CTA...... .T........ .....G.... .......... .......... .......... .......... ......G... 320
 HAP3_DLOOP ☐  .CTA...... .T........ .....G.... .......... .......... .......... .......... ......G... 320
 HAP4_DLOOP ☐  .CTA...... .T........ .....-.... .......... .......... .......... .......... ......G... 319
  Consensus    ATTAAACCCA ACCAAGTAAC AATAA-AACT TAGACAGACA TAAACTGCAA ACAGAATACT CACAAAGAAC TCCAACGCAG
```

Conservation

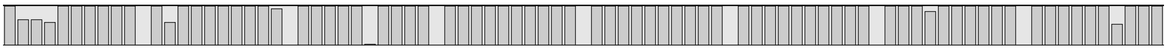

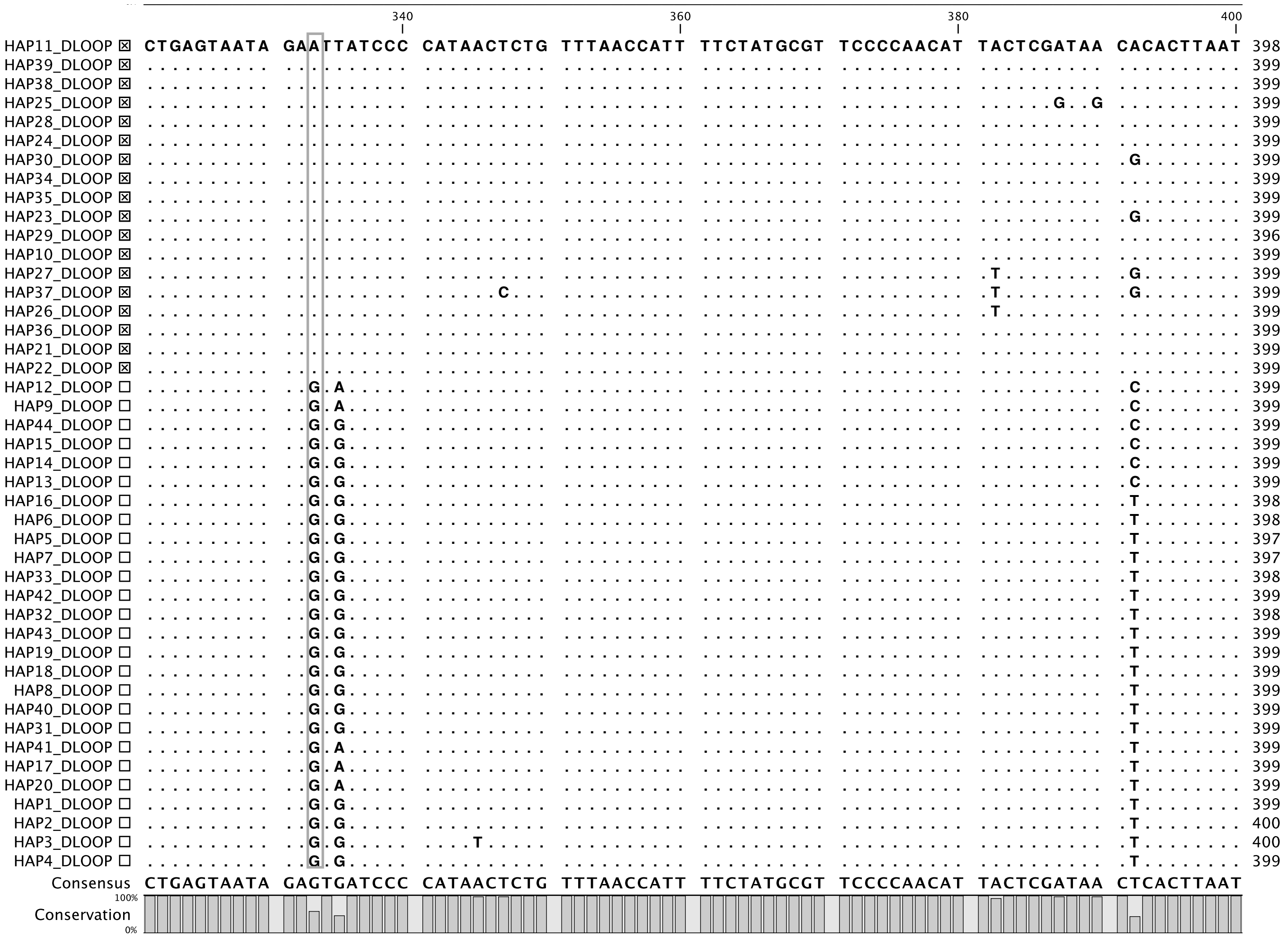
340
360
380
400
HAP11_DLOOP CTGAGTAATA GAATTATCCC CATAACTCTG TTTAACCATT TTCTATGCGT TCCCCAACAT TACTCGATAA CACACTTAAT 398
Consensus CTGAGTAATA GAGTGATCCC CATAACTCTG TTTAACCATT TTCTATGCGT TCCCCAACAT TACTCGATAA CTCACTTAAT
Conservation
100%
0%

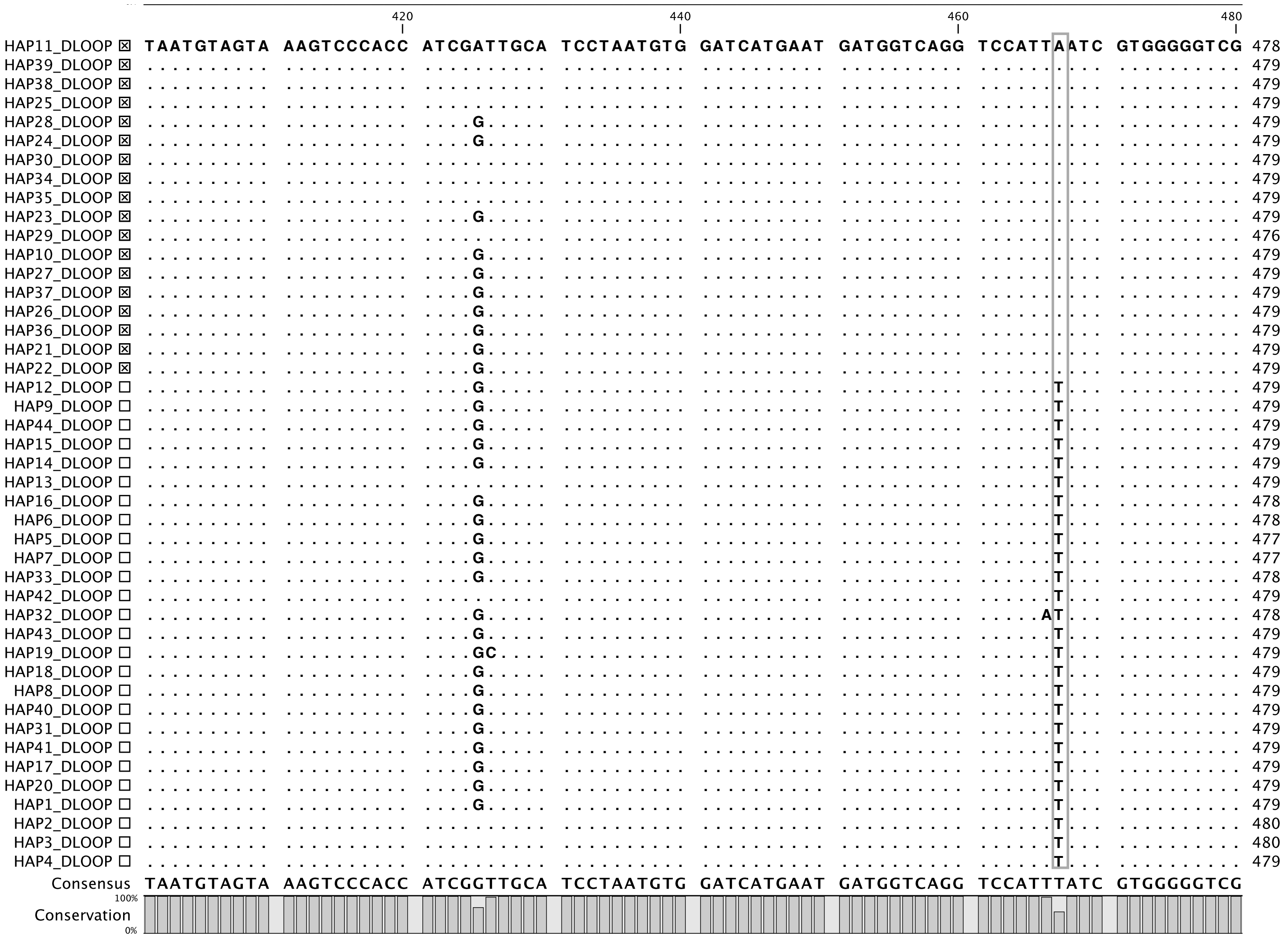

420 440 460 480
HAP11_DLOOP ☒ TAATGTAGTA AAGTCCCACC ATCGATTGCA TCCTAATGTG GATCATGAAT GATGGTCAGG TCCATTAATC GTGGGGGTCG 478
HAP39_DLOOP ☒ 479
HAP38_DLOOP ☒ 479
HAP25_DLOOP ☒ 479
HAP28_DLOOP ☒ ....G..... 479
HAP24_DLOOP ☒ ....G..... 479
HAP30_DLOOP ☒ 479
HAP34_DLOOP ☒ 479
HAP35_DLOOP ☒ 479
HAP23_DLOOP ☒ ....G..... 479
HAP29_DLOOP ☒ 476
HAP10_DLOOP ☒ ....G..... 479
HAP27_DLOOP ☒ ....G..... 479
HAP37_DLOOP ☒ ....G..... 479
HAP26_DLOOP ☒ ....G..... 479
HAP36_DLOOP ☒ ....G..... 479
HAP21_DLOOP ☒ ....G..... 479
HAP22_DLOOP ☒ ....G..... 479
HAP12_DLOOP ☐ ....G..... ....T... 479
HAP9_DLOOP ☐ ....G..... ....T... 479
HAP44_DLOOP ☐ ....G..... ....T... 479
HAP15_DLOOP ☐ ....G..... ....T... 479
HAP14_DLOOP ☐ ....G..... ....T... 479
HAP13_DLOOP ☐ ....T... 479
HAP16_DLOOP ☐ ....G..... ....T... 478
HAP6_DLOOP ☐ ....G..... ....T... 478
HAP5_DLOOP ☐ ....G..... ....T... 477
HAP7_DLOOP ☐ ....G..... ....T... 477
HAP33_DLOOP ☐ ....G..... ....T... 478
HAP42_DLOOP ☐ ....T... 479
HAP32_DLOOP ☐ ....G..... ....AT... 478
HAP43_DLOOP ☐ ....G..... ....T... 479
HAP19_DLOOP ☐ ....GC.... ....T... 479
HAP18_DLOOP ☐ ....G..... ....T... 479
HAP8_DLOOP ☐ ....G..... ....T... 479
HAP40_DLOOP ☐ ....G..... ....T... 479
HAP31_DLOOP ☐ ....G..... ....T... 479
HAP41_DLOOP ☐ ....G..... ....T... 479
HAP17_DLOOP ☐ ....G..... ....T... 479
HAP20_DLOOP ☐ ....G..... ....T... 479
HAP1_DLOOP ☐ ....G..... ....T... 479
HAP2_DLOOP ☐ ....T... 480
HAP3_DLOOP ☐ ....T... 480
HAP4_DLOOP ☐ ....T... 479
Consensus TAATGTAGTA AAGTCCCACC ATCGGTTGCA TCCTAATGTG GATCATGAAT GATGGTCAGG TCCATTTATC GTGGGGGTCG
100%
Conservation
0%

```
                              500                  520                  540                  560
                                |                    |                    |                    |
HAP11_DLOOP ☒ CACAGAATGA ATTATTTCTG GCCTCTGGTT CCTTCGTCAG GTTCCCTTAT CACATAAACC CCCCATCAAT CGCACTTTTG 558
HAP39_DLOOP ☒ .......... .......... .......... .......... .......... .......... .......... .......... 559
HAP38_DLOOP ☒ .......... .......... .......... .......... .......... .......... .......... .......... 559
HAP25_DLOOP ☒ .......... .......... .......... .......... .......... .......... ..A....... .......... 559
HAP28_DLOOP ☒ .......... .......... .......... .......... .......... .......... .......... .......... 559
HAP24_DLOOP ☒ .......... .......... ..-....... .......... .......... .......... .......... .......... 558
HAP30_DLOOP ☒ .......... .......... .......... .......... .......... .......... .......... .......... 559
HAP34_DLOOP ☒ .......... .......... .......... .......... .......... .......... .......... .......... 559
HAP35_DLOOP ☒ .......... .......... ..-....... .......... .......... .......... .......... .......... 558
HAP23_DLOOP ☒ .......... .......... .......... .......... .......... .......... .......... .......... 559
HAP29_DLOOP ☒ .......... .......... .......... .......... .......... .......... .......... .......... 556
HAP10_DLOOP ☒ .......... .......... ..-....... .........A -......... .......... .......... .......... 557
HAP27_DLOOP ☒ .......... .......... .......... .......... .......... .......... .......... .......... 559
HAP37_DLOOP ☒ .......... .......... .......... .......... .......... .......... .......... .......... 559
HAP26_DLOOP ☒ .......... .......... .......... .......... .......... .......... .......... .......... 559
HAP36_DLOOP ☒ .......... .......... .......... .......... .......... .......... .......... .......... 559
HAP21_DLOOP ☒ .......... .......... .......... .......... .......... .......... .......... .......... 559
HAP22_DLOOP ☒ .......... .......... .......... .......... .......... .......... .......... .......... 559
HAP12_DLOOP ☐ .......... .......... ........A. .......... .......... .......... ..A....G.. .......... 559
 HAP9_DLOOP ☐ .......... .......... ..-....... .........A -......... .......... ..A....... .......... 557
HAP44_DLOOP ☐ .......... .......... .......... .......... .......... .......... ..A....... .......... 559
HAP15_DLOOP ☐ .......... .......... .......... .......... .......... .......... ..A....... .......... 559
HAP14_DLOOP ☐ .......... .......... .......... .......... .......... .......... ..A....... .......... 559
HAP13_DLOOP ☐ .......... .......... .......... .......... .......... .......... ..A....G.. .......... 559
HAP16_DLOOP ☐ .......... .......... .......... .......... .......... .......... ..A....... .......... 558
 HAP6_DLOOP ☐ .......... .......... .......... .......... .......... .......... ..A....... .......... 558
 HAP5_DLOOP ☐ .......... .......... .......... .......... .......... .......... ..A....G.. .......... 557
 HAP7_DLOOP ☐ .......... .......... .......... .......... .......... .......... ..A....... .......... 557
HAP33_DLOOP ☐ .......... .......... .......... .......... .......... .......... ..A....... .......... 558
HAP42_DLOOP ☐ .......... .......... .......... .......... .......... .......... ..A.....G. ........C. 559
HAP32_DLOOP ☐ .......... .......... .......... .......... .......... .......... ..A....... .......... 558
HAP43_DLOOP ☐ .......... .......... .......... .......... .......... .......... ..A....... .......... 559
HAP19_DLOOP ☐ .......... .......... .......... .......... .......... .......... ..A.....G. ........C. 559
HAP18_DLOOP ☐ .......... .......... .......... .......... .......... .......... ..A.....G. ........C. 559
 HAP8_DLOOP ☐ .......... .......... ..-....... .......... .......... .......... ..A.....G. ........C. 558
HAP40_DLOOP ☐ .......... .......... .......... .......... .......... .......... ..A.....G. ........C. 559
HAP31_DLOOP ☐ .......... .......... .......... .......... .......... .......... ..A.....G. ........C. 559
HAP41_DLOOP ☐ .......... .......... ..-....... .......... .......... .......... ..A.....G. .CG.....C. 558
HAP17_DLOOP ☐ .......... .......... .......... .......... .......... .......... ..A.....G. ........C. 559
HAP20_DLOOP ☐ .......... .......... .......... .......... .......... .......... ..A....G.. .......... 559
 HAP1_DLOOP ☐ .......... .......... .......... .......... .......... .......... ..A.....G. ........C. 559
 HAP2_DLOOP ☐ .......... .......... .......... .......... .......... .......... ..A.....G. ........C. 560
 HAP3_DLOOP ☐ .......... .......... .......... .......... .......... .......... ..A.....G. ........C. 560
 HAP4_DLOOP ☐ .......... .......... .......... .......... .......... .......... ..A.....G. ........C. 559
  Consensus   CACAGAATGA ATTATTTCTG GCCTCTGGTT CCTTCGTCAG GTTCCCTTAT CACATAAACC CCACATCAAT CGCACTTTTG
```

Conservation 100% 0%

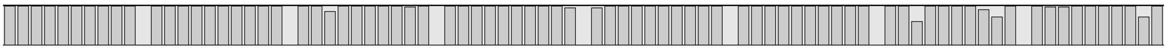

```
HAP11_DLOOP ☒ CGCATAAGTT AATGCAT 575
HAP39_DLOOP ☒ .......... ....... 576
HAP38_DLOOP ☒ .......... ....... 576
HAP25_DLOOP ☒ .......... ....... 576
HAP28_DLOOP ☒ .......... ....... 576
HAP24_DLOOP ☒ .......... ....... 575
HAP30_DLOOP ☒ .......... ....... 576
HAP34_DLOOP ☒ .......... ....... 576
HAP35_DLOOP ☒ .......... ....... 575
HAP23_DLOOP ☒ .......... ....... 576
HAP29_DLOOP ☒ .......... ....... 573
HAP10_DLOOP ☒ .......... ....... 574
HAP27_DLOOP ☒ .......... ....... 576
HAP37_DLOOP ☒ .......... ....... 576
HAP26_DLOOP ☒ .......... ....... 576
HAP36_DLOOP ☒ .......... ....... 576
HAP21_DLOOP ☒ .......... ....... 576
HAP22_DLOOP ☒ .......... ....... 576
HAP12_DLOOP ☐ .......... ....... 576
 HAP9_DLOOP ☐ .......... ....... 574
HAP44_DLOOP ☐ .......... ....... 576
HAP15_DLOOP ☐ .......... ....... 576
HAP14_DLOOP ☐ .......... ....... 576
HAP13_DLOOP ☐ .......... ....... 576
HAP16_DLOOP ☐ .......... ....... 575
 HAP6_DLOOP ☐ .......... ....... 575
 HAP5_DLOOP ☐ .......... ....... 574
 HAP7_DLOOP ☐ .......... ....... 574
HAP33_DLOOP ☐ .......... ....... 575
HAP42_DLOOP ☐ .......... ....... 576
HAP32_DLOOP ☐ .......... ....... 575
HAP43_DLOOP ☐ .......... ....... 576
HAP19_DLOOP ☐ .......... ....... 576
HAP18_DLOOP ☐ .......... ....... 576
 HAP8_DLOOP ☐ .......... ....... 575
HAP40_DLOOP ☐ .......... ....... 576
HAP31_DLOOP ☐ .......... ....... 576
HAP41_DLOOP ☐ .......... ....... 575
HAP17_DLOOP ☐ .......... ....... 576
HAP20_DLOOP ☐ .......... ....... 576
 HAP1_DLOOP ☐ .......... ....... 576
 HAP2_DLOOP ☐ .......... ....... 577
 HAP3_DLOOP ☐ .......... ....... 577
 HAP4_DLOOP ☐ .......... ....... 576
  Consensus   CGCATAAGTT AATGCAT
```

Conservation

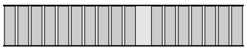

Supplement: S2 File — Marked haplotypes belong to haplogroup A. (PDF) [file pone.0143297.s002.pdf]
