# Supplementary material for: A COI Nonsynonymous Mutation as Diagnostic Tool for Intraspecific Discrimination in the European Anchovy Engraulis encrasicolus (Linnaeus)
Source: PLoS One. 2015 Nov 24;10(11):e0143297. doi: 10.1371/journal.pone.0143297 (PMC4657973; doi:10.1371/journal.pone.0143297)

```
                                        20                  40                  60                  80
                                         |                   |                   |                   |
HAP8_COI ☒   CTATATCTTA TTTTCGGTGC CTGAGCAGGA -ATGGTAGGG ACAGCACTTA GCCTCCTTAT TCGAGCAGAA CTAAGCCAAC 79
HAP9_COI ☒   .......... .......... .......... -......... .......... .......... .......... ..G....... 79
HAP22_COI ☒  .......... .......... .......... -......... .......... .......... .T........ .......... 79
HAP23_COI ☒  .......... .......... .......... -......... .......... .......... .T........ .......... 79
HAP17_COI ☒  .......... ....T..... .......... -......... .......... .......... .......... .......... 79
HAP16_COI ☒  .......... .......... .......... -......... .......... .......... .......... .......... 79
HAP21_COI ☒  .......... .......... .....G.... -......... .......... .......... .......... .......... 79
HAP24_COI ☒  .......... .......... .......... -......... .......... .......... .......... .......... 79
HAP15_COI ☒  .......... .......... .......... -......... .......... .......... .......... .......... 79
HAP10_COI ☒  .......... .......... .......... -......... .......... .......... .......... .......... 79
HAP18_COI ☒  .......... .......... .......... -......... .......... .......... .......... .......... 79
HAP25_COI ☒  .......... .......... .......... -......... .......... .......... .......... .......... 79
HAP38_COI ☒  .......... .......... .......... -......... .......... .......... .......... .......... 79
HAP36_COI ☒  .......... .......... .......... -......... .......... .......... .......... .......... 79
HAP37_COI ☒  .......... .......... .......... -......... .......... .......... .......... .......... 79
HAP11_COI ☒  .......... .......... .......... -......... .......... .......... .......... .......... 79
HAP20_COI ☒  .......... .......... .......... -......... .......... .......... .......... ....A..... 79
HAP26_COI ☒  .......... .......... .......... -......... .......... .......... .......... .......... 79
HAP39_COI ☒  .......... .......... .......... -......... .......... .......... .......... .......... 79
HAP19_COI ☒  .......... .......... .......... -......... .......... .......... .......... .......... 79
HAP27_COI ☒  .......... .......... .......... -......... .......... .......... .......... .......... 79
HAP1_COI ☐   .......... .......... .......... -......... .......... .......... .......... .......... 79
HAP30_COI ☐  .......... .......... .......... -......... .......... .......... .......... .......... 79
HAP14_COI ☐  .......... .......... .......... -......... .......... .......... .......... .......... 79
HAP32_COI ☐  .......... .......... T......... -......... .......... .......... .......... .......... 79
HAP31_COI ☐  .......... .......... .......... -......... .......... .......... .......... .......... 79
HAP3_COI ☐   .......... .......... .......... -......... .......... .......... .......... .......... 79
HAP4_COI ☐   .......... .......... .......... -......... .......... .......... .......... .......... 79
HAP33_COI ☐  .......... .......... .......... -......... .......... .......... .......... .......... 79
HAP34_COI ☐  .......... .......... .......... -......... .......... .......... .......... .......... 79
HAP2_COI ☐   .......... .......... .......... -......... .......... .......... .......... .......... 79
HAP6_COI ☐   .......... .......... .......... -......... .......... .......... .......... .......... 79
HAP28_COI ☐  .......... .......... .......... C.......C. .-........ .......... .....G.... .......... 79
HAP7_COI ☐   .......... .......... .......... -......... .......... .......... .......... .......... 79
HAP35_COI ☐  .......... .......... .......... -......... .......... .......... .......... .......... 79
HAP5_COI ☐   .......... .......... .......... -......... .......... .......... .......... .......... 79
HAP29_COI ☐  .......... .......... .......... -......... .......... .......... .......... .......... 79
HAP12_COI ☐  .......... .......... .......... -......... .......... .......... .......... .......... 79
HAP13_COI ☐  .......... .......... .......... -......... G......... .......... .......... .......... 79
```

Conservation 100% 0%

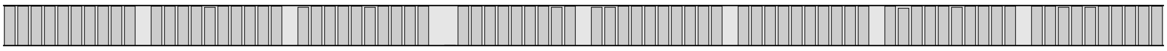

```
                                       100                 120                 140                 160
                                         |                   |                   |                   |
HAP8_COI ☒   CAGGAGCACT TCTGGGGGAC GATCAAATTT ATAACGTAAT CGTTACTGCT CACGCATTCG TAATAATCTT TTTCATGGTA 159
HAP9_COI ☒   .......... .......... .......... .......... .......... .......... .......... .......... 159
HAP22_COI ☒  .......... .......... .......... .......... .......... .......... .......... .......... 159
HAP23_COI ☒  .......... .......... .......... .......... .......... .......... .......... .......... 159
HAP17_COI ☒  .......... .......... .......... .......... .......... .......... .......... ......A... 159
HAP16_COI ☒  .......... .......... .......... .......... .......... .......... .......... .......... 159
HAP21_COI ☒  .......... .......... .......... .......... .......... .......... .......... .......... 159
HAP24_COI ☒  .......... .......... .......... .......... .......... .......... .......... .......... 159
HAP15_COI ☒  .......... .......... .......... .......... .......... .......... .......... .......... 159
HAP10_COI ☒  .......... .......... .......... .C........ .......... .......... .......... .......... 159
HAP18_COI ☒  .......... .......... .......... .......... .......... .......... .......... .......... 159
HAP25_COI ☒  .......... .......... .......... .......... .......... .......... .......... .......... 159
HAP38_COI ☒  .......... .......... .......... .......... .......... .......... .......... .......... 159
HAP36_COI ☒  .......... .......... .......... .......... .......... .......... .......... .......... 159
HAP37_COI ☒  .......... .......... .......... .......... ......C... .......... .......... .......... 159
HAP11_COI ☒  .......... .......... .......... .......... .......... .......... .......... .......... 159
HAP20_COI ☒  .......... .......... .......... .......... ....T.C... .......... .......... .......... 159
HAP26_COI ☒  .......... .......... .......... .......... .......... .......... .......... .......... 159
HAP39_COI ☒  .G........ .......... .......... .......... .......... .......... .......... .......... 159
HAP19_COI ☒  .......... .......... .......... .......... .......... .......... .......... .......... 159
HAP27_COI ☒  .......... .......... .......... .......... .......... .......... .......... .......... 159
HAP1_COI ☐   .......... .......... .......... .......... ......C... .......... .......... ...T...... 159
HAP30_COI ☐  .......... .......... .......... .......... ......C... .......... .......... .......... 159
HAP14_COI ☐  .......... .......... .......... .......... ....T.C... .......... .......... .......... 159
HAP32_COI ☐  .......... .......... .......... .......... ....TAC... .......... .......... .......... 159
HAP31_COI ☐  .......... .......... .......... .......... ....T.C... .......... .......... .......... 159
HAP3_COI ☐   .......... .......... .......... .......... ......C... .......... .......... .......... 159
HAP4_COI ☐   .......... .......... .......... .......... .......... .......... .......... .......... 159
HAP33_COI ☐  .......... .......... .......... .......... .......... .......... .......... .......... 159
HAP34_COI ☐  .......... .......... .......... .......... .......... .......... .......... .......... 159
HAP2_COI ☐   .......... .......... .......... .......... ......C... .......... .......... .......... 159
HAP6_COI ☐   .......... .......... .......... .......... .......... .......... .......... .......... 159
HAP28_COI ☐  .......... .......... .......... .......... .......... .......... .......... .......... 159
HAP7_COI ☐   .......... .......... .......... .......... .......... ..T....... .......... .......... 159
HAP35_COI ☐  .......... .....A.... .......... .......... .......... .......... .......... .......... 159
HAP5_COI ☐   .......... .......... .......... .......... .......... .......... .......... .......... 159
HAP29_COI ☐  .......... .......... .......... .......... .......... .......... .......... .......... 159
HAP12_COI ☐  .......... .......... .......... .......... .......... .......... .......... .......... 159
HAP13_COI ☐  .......... .......... .......... .......... .......... .......... .......... .......... 159
```

Conservation 100% 0%

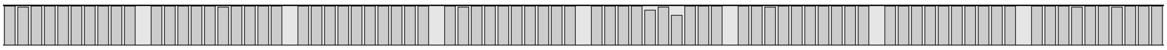

```
                     180                 200                 220                 240
                       |                   |                   |                   |
HAP8_COI ☒  ATGCCCATTC TAATCGGTGG GTTCGGGAAT TGACTAGTTC CTCTTATACT AGGGGCCCCA GACATGGCAT TCCCCCGAAT 239
HAP9_COI ☒  .......... .......... .......... .......... .......... .......... .......... .......... 239
HAP22_COI ☒ .......... .......... .......... .......... .......... .......... .......... .......... 239
HAP23_COI ☒ .......... .......... .......... .......... .......... .......... .......... .......... 239
HAP17_COI ☒ .......... .......... .......... .......... .......... .......... .......... .......... 239
HAP16_COI ☒ .......... .......... .......... ........C. .......... ..A....... .......... .......... 239
HAP21_COI ☒ .......... .......... .......... .......... .......... .......... ..T....... .......... 239
HAP24_COI ☒ .......... .......... .......... .......... .......... .......... .......... .......... 239
HAP15_COI ☒ .......... .......... .......... .......... .......... .......... .......... .......... 239
HAP10_COI ☒ .......... .......... .......... .......... .......... .......... .......... .......... 239
HAP18_COI ☒ .......... .......... .......... .......... .......... .......... .......... .......... 239
HAP25_COI ☒ .......... .......... .......... .......... .......... .......... .......... .......... 239
HAP38_COI ☒ .......... .......... .......... .......... .......... .......... .......... .......... 239
HAP36_COI ☒ .......... .......... .......... .......... .......... .......... .......... .......... 239
HAP37_COI ☒ .......... .......... .......... .......... .......... .......... .......... .......... 239
HAP11_COI ☒ .......... .......... .......... .......... .......... .......... .......... .......... 239
HAP20_COI ☒ .......... .......... .......... .......... .......... .......... .......... .......... 239
HAP26_COI ☒ .......... .......... .......... .......... .......... .......... .......... .......... 239
HAP39_COI ☒ .......... .......... .......... .......... .......... .......... .......... .......... 239
HAP19_COI ☒ .......... .......... .......... .......... .......... .......... .......... .......... 239
HAP27_COI ☒ .......... .......... .......... .......... .......... .......... .......... .......... 239
HAP1_COI ☐  .......... .......... .......... .......... .......... .......... .......... .......... 239
HAP30_COI ☐ .......... .......... .......... .......... .......... .......... .......... .......... 239
HAP14_COI ☐ .......... .......... .......... .......... .......... .......... .......... .......... 239
HAP32_COI ☐ .......... .......... .......... .......... .......... .......... .......... .......... 239
HAP31_COI ☐ .......... .......... .......... .......... .......... .......... .......... .......... 239
HAP3_COI ☐  .......... .......... .......... .......... .......... .......... .......... .......... 239
HAP4_COI ☐  .......... .......... .......... .......... .......... .......... .......... .......... 239
HAP33_COI ☐ .......... .......... .......... .......... .......... .......... .......... .......... 239
HAP34_COI ☐ .......... .......... .......... .......... .......... .......... .......... .......... 239
HAP2_COI ☐  .......... .......... .......... .......... .......... .......... .......... .......... 239
HAP6_COI ☐  .......... .......... .......... .......... .......... G......... .......... .......... 239
HAP28_COI ☐ .......... .......... .......... .......... .......... .......... .......... .......... 239
HAP7_COI ☐  .......... .......... .......... .......... .......... .......... .......... .......... 239
HAP35_COI ☐ .......... .......... .......... .......... .......... .......... .......... .......... 239
HAP5_COI ☐  .......... .......... .......... .......... .......... .......... .......... .......... 239
HAP29_COI ☐ .......... .......... .......... .......... .......... .......... .......... .......... 239
HAP12_COI ☐ .......... .......... .......... .......... .......... .......... .......... .......... 239
HAP13_COI ☐ ..A....... .......... .......... .......... .......... .......... .......... .......... 239
```

100%
Conservation
0%

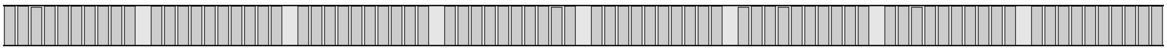

```
                     260                 280                 300                 320
                       |                   |                   |                   |
HAP8_COI ☒  GAACAATATG AGCTTTTGAC TCCTTCCCCC TTCTTTCCTT CTCCTCTTAG CATCATCTGG TGTTGAAGCA GGGGCCGGGA 319
HAP9_COI ☒  .......... .......... .......... .......... .......... .......... .......... .......... 319
HAP22_COI ☒ .......... .......... .......... .......... .......... .G........ .......... .......... 319
HAP23_COI ☒ .......... .......... .......... .......... .......... .......... .......... .......... 319
HAP17_COI ☒ .......... .......... .......... .......... .......... .......... .......... .......... 319
HAP16_COI ☒ .......... .......... .......... .......... .......... .......... .......... .......... 319
HAP21_COI ☒ .......... .......... .......... .......... .......... .......... .......... .......... 319
HAP24_COI ☒ .......... .......... .......... .......... .......... .......... .......... .......... 319
HAP15_COI ☒ .......... .......... .......... .......... .......... .......... .......... .......... 319
HAP10_COI ☒ .......... .......... .......... .......... .......... .......... .......... .......... 319
HAP18_COI ☒ .......... .......... .......... .......... .......... .......... .......... .......... 319
HAP25_COI ☒ .......... .......... .......... .......... .......... .......... .......... .......... 319
HAP38_COI ☒ .......... .......... .......... .......... .......... .......... .......... .......... 319
HAP36_COI ☒ .......... .......... .......... .......... .......... .......... .......... .......... 319
HAP37_COI ☒ .......... .......... .......... .......... .......... .......... .......... .......... 319
HAP11_COI ☒ .......... .......... .......... .......... .......... .......... .......... .......... 319
HAP20_COI ☒ .......... .......... .......... .......... .......... .......... .......... .......... 319
HAP26_COI ☒ .......... .......... .......... .......... .......... .......... .......... .......... 319
HAP39_COI ☒ .......... .......... .......... .......... ........G. .......... .......... .......... 319
HAP19_COI ☒ ......C... .......... .......... .......... .......... .......... .......... .......... 319
HAP27_COI ☒ .......... .......... .......T.. .......... .......... .......... .......... ..A....... 319
HAP1_COI ☐  .......... .......... .......T.. .......... .......... .......... .......... ..A....... 319
HAP30_COI ☐ .......... .......... .......T.. .......... .......... .......... .......... ..A....... 319
HAP14_COI ☐ .......... .......... .......T.. .......... .......... .......... .......... ..A....... 319
HAP32_COI ☐ .......... .......... .......T.. .......... .......... .......... .......... ..A....... 319
HAP31_COI ☐ .......... .......... .......T.. .......... .......... .......... .......... ..A....... 319
HAP3_COI ☐  .......... .......... .......T.. .......... .......... .......... .......... ..A....... 319
HAP4_COI ☐  .......... .......... .......T.. .......... .......... .......... .......... ..A....... 319
HAP33_COI ☐ .......... .......... .......T.. .......... .......... .......... .......... ..A.....A. 319
HAP34_COI ☐ .......... .......... .......T.. .......... .......... .......... .......... ..A.....A. 319
HAP2_COI ☐  .......... .......... .......T.. .......... .......... .......... .......... ..A.....A. 319
HAP6_COI ☐  .......... .......... .......T.. .......... .......... .......... .......... ..A....... 319
HAP28_COI ☐ .......... .......... .......T.. .......... .......... .......... .......... ..A....... 319
HAP7_COI ☐  .......... .......... .......T.. .......... .......... .......... .......... ..A....... 319
HAP35_COI ☐ .......... .......... .......T.. .......... .......... .......... .......... ..A....... 319
HAP5_COI ☐  .......... .......... .......T.. .......... .......... .......... .......... ..A....... 319
HAP29_COI ☐ .......... .......... .......T.. .......... .......... .......... .......... ..A....... 319
HAP12_COI ☐ .......... .......... .......T.. .......... .......... .......... .......... .......... 319
HAP13_COI ☐ .......... .......... .......T.. .......... .......... .......... .......... ..A....... 319
```

100%
Conservation
0%

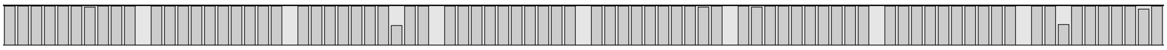

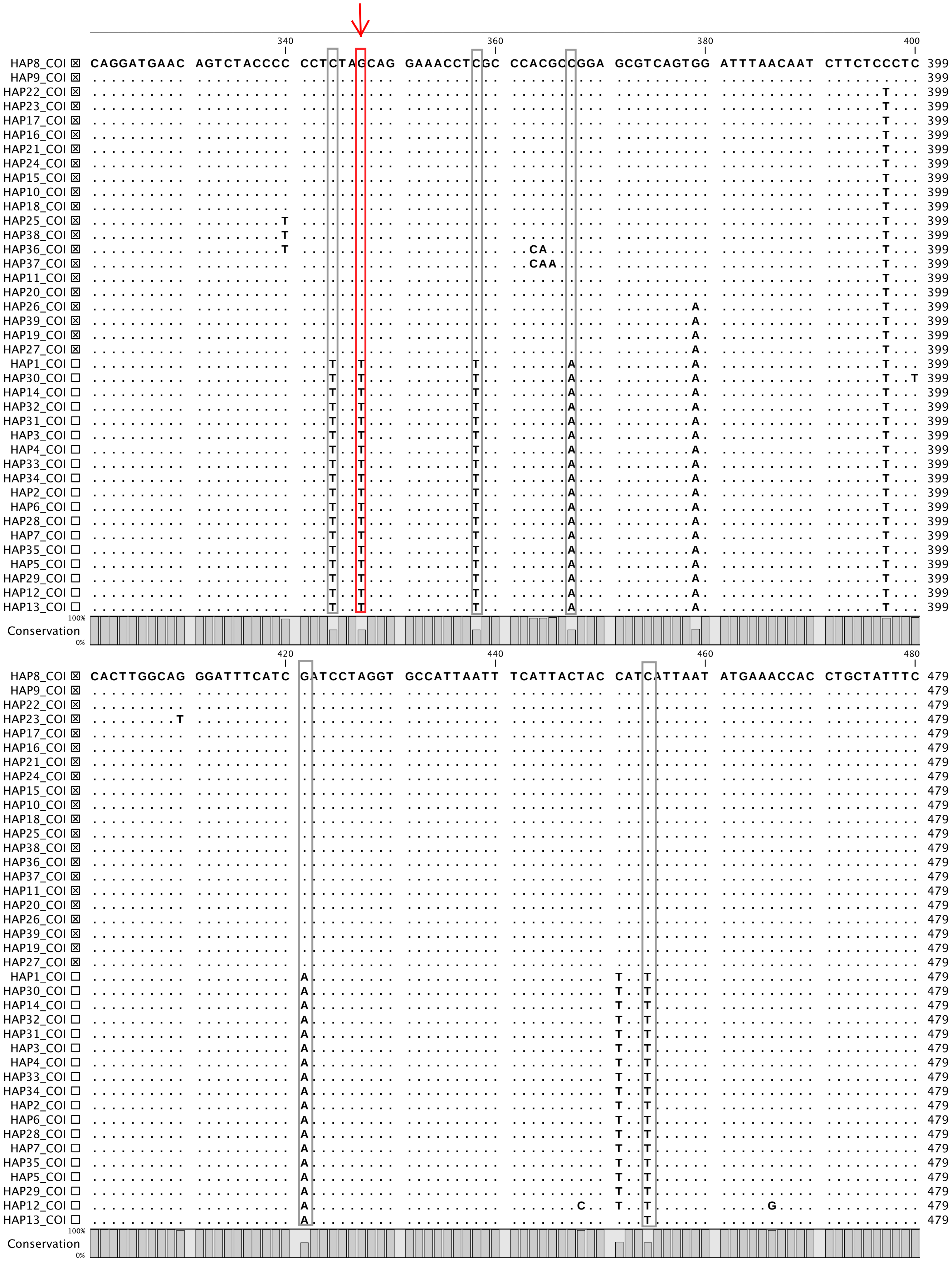
HAP8_COI CAGGATGAAC AGTCTACCCC CCTCTAGCAG GAAACCTCGC CCACGCCGGA GCGTCAGTGG ATTTAACAAT CTTCTCCCTC 399
HAP8_COI CACTTGGCAG GGATTTCATC GATCCTAGGT GCCATTAATT TCATTACTAC CATCATTAAT ATGAAACCAC CTGCTATTTC 479
Conservation

```
                                          500                 520                 540                 560
HAP8_COI  ☒ ACAATACCAG ACACCTCTAT TTGTCTGAGC TGTATTAATC ACGGCAGTAC TTTTACTTCT TTCACTACCC GTTCTAGCTG 559
HAP9_COI  ☒ .......... .......... .......... .......... .......... .......... .......... .......... 559
HAP22_COI ☒ G......... .......... .......... .......... .......... .......... .......... .......... 559
HAP23_COI ☒ .......... .......... .......... .......... .......... .......... .......... .......... 559
HAP17_COI ☒ .......... .......... .......... ...G...... .......... .......... .......... .......... 559
HAP16_COI ☒ .......... .......... .......... .......... .......... .......... .......... .......... 559
HAP21_COI ☒ .......... .......... .......... .......... .......... .......... .......... .......... 559
HAP24_COI ☒ .......... .......... .......... .......... .......... .......... .......... .......... 559
HAP15_COI ☒ .......... .......... .......... .......... .......... .......... ......G... .......... 559
HAP10_COI ☒ .......... .......... .......... .......... .......... .......... .......... .......... 559
HAP18_COI ☒ .......... .......... .......... .......... .......... .......... .......... .......... 559
HAP25_COI ☒ .......... .......... .......... .......... .......... .......... .......... .......... 559
HAP38_COI ☒ .......... .......... .......... .......... .......... .......... .......... .......... 559
HAP36_COI ☒ .......... .......... .......... .......... .......... .......... .......... .......... 559
HAP37_COI ☒ ...G...... .......... .......... .......... .......... .......... .......... .......... 559
HAP11_COI ☒ .......... .......... .......... .......... .......... .......... .......... .......... 559
HAP20_COI ☒ .......... .......... .......... .......... .......... .......... .......... .......... 559
HAP26_COI ☒ .......... .......... .......... .......... .......... .......... .......... .......... 559
HAP39_COI ☒ .......... .......... .......... .......... .......... .......... .......... .......... 559
HAP19_COI ☒ .......... .......... .......... .......... .......... .......... .......... .......... 559
HAP27_COI ☒ .......... .......... .......... .......... .......... .......... .......... .......... 559
HAP1_COI  ☐ .......... .......... .......... .......... .......... .......... .......... .......... 559
HAP30_COI ☐ .......... .......... .......... .......... .......... .......... .......... .......... 559
HAP14_COI ☐ .......... .......... .......... .......... .......... .......... .......... .......... 559
HAP32_COI ☐ .......... .......... .......... .......... .......... .......... .......... .......... 559
HAP31_COI ☐ .......... .......... .......... .......... .......... .......... .......... .......... 559
HAP3_COI  ☐ .......... .......... .......... .......... .......... .......... .......... .......... 559
HAP4_COI  ☐ .......... .......... .......... .......... .......... .......... .......... .......... 559
HAP33_COI ☐ .......... .......... .......... .......... .......... .......... .......... .......... 559
HAP34_COI ☐ .......... .......... .......... .......... .......... .......... .......... .......... 559
HAP2_COI  ☐ .......... .......... .......... .......... .......... .......... .......... .......... 559
HAP6_COI  ☐ .......... .......... .......... .......... .......... .......... .......... .......... 559
HAP28_COI ☐ .......... .......... .......... .......... .......... .......... .......... .......... 559
HAP7_COI  ☐ .......... .......... .......... .......... .......... .......... .......... .......... 559
HAP35_COI ☐ .......... .......... .......... .......... .......... .......... .......... .......... 559
HAP5_COI  ☐ .......... .......... .......... .......... .......... .......... .......... .......... 559
HAP29_COI ☐ .......... .......... .......... .......... .......... .......... .......... .......... 559
HAP12_COI ☐ .......... .......... .......... .......... .......... .......... .......... .......... 559
HAP13_COI ☐ .......... .......... .......... .......... .......... .......... .......... .......... 559
100%
Conservation
0%
                                          580                 600                 620                 640
HAP8_COI  ☒ CTGGGATTAC TATGCTTCTT ACAGACCGGA ACCTAAATAC TACTTTCTTC GACCCAGCAG GGGGAGGAGA CCCAATTCTT 639
HAP9_COI  ☒ .......... .......... ........A. .......... .......... .......... .......... .......... 639
HAP22_COI ☒ .......... .......... ........A. .......... .......... .......... .......... .......... 639
HAP23_COI ☒ .......... .......... ........A. .......... .......... .......... .......... .......... 639
HAP17_COI ☒ .......... .......... ........A. .......... .......... .......... .......... .......... 639
HAP16_COI ☒ .......... .......... ........A. .......... .......... .......... .......... .......... 639
HAP21_COI ☒ .......... .......... ........A. .......... .......... .......... .......... .......... 639
HAP24_COI ☒ .......... .......... ........A. .......... .......... ........G. .......... .......... 639
HAP15_COI ☒ .......... .......... ........A. .......... .......... .......... .......... .......... 639
HAP10_COI ☒ .......... .......... ........A. .......... .......... .......... .......... .......... 639
HAP18_COI ☒ .......... .......... ........A. .......... .......... .......... .......... .......... 639
HAP25_COI ☒ .......... .......... ........A. .......... .......... .......... .......... .......... 639
HAP38_COI ☒ .......... .......... .....T..A. .......... .......... .......... .......... .......... 639
HAP36_COI ☒ .......... .......... ........A. .......... .......... .......... .......... .......... 639
HAP37_COI ☒ .......... .......... ........A. .......... .......... .......... .......... .......... 639
HAP11_COI ☒ .......... .......... ........A. .......... .......... .......... .......... ...T...... 639
HAP20_COI ☒ .......... .......... ........A. .......... .......... .......... .......... ...G...... 639
HAP26_COI ☒ .......... .......... ........A. .......... .......... .......... .......... .......... 639
HAP39_COI ☒ .......... .......... ........A. .......... .......... .......... .......... .......... 639
HAP19_COI ☒ .......... .......... ........A. .......... .......... .......... .......... .......... 639
HAP27_COI ☒ .......... .......... ........A. .......... .......... .......... .......... .......... 639
HAP1_COI  ☐ .......... .......... ........A. .......... .......... .......... .......... ...G...... 639
HAP30_COI ☐ .......... .......... ........A. .......... .......... ....-..... .......... ...G...... 638
HAP14_COI ☐ .......... .......... ....T...A. .......... .......... T......... .......... ...G...... 639
HAP32_COI ☐ .......... .......... ........A. .......... .......... .......... .......... ...G...... 639
HAP31_COI ☐ .......... .......... ........A. .......... .......... .......... .......... ...G...... 639
HAP3_COI  ☐ .......... .......... ........A. .......... .......... .....G.... .......... ...G...... 639
HAP4_COI  ☐ .......... .......... ........A. .......... .......... .....G.... .......... ...G...... 639
HAP33_COI ☐ .......... .......... ........A. .......... .......... .......... .......... ...G...... 639
HAP34_COI ☐ .......... .......... ........A. ....G..... .......... .......... .......... ...G...... 639
HAP2_COI  ☐ .......... .......... ........A. .......... .......... .......... .......... ...G...... 639
HAP6_COI  ☐ .......... .......... ........A. .......... .......... .......... .......... ...G...... 639
HAP28_COI ☐ .......... .......... ........A. .......... .......... .......... .......... ...G...... 639
HAP7_COI  ☐ .......... .......... ........A. .......... .......... .......... .......... ...G...... 639
HAP35_COI ☐ .......... .......... ........A. .......... .......... .......... .......... ...G...... 639
HAP5_COI  ☐ .......... .......... ........A. .......... .......... .......... .......... ...G...... 639
HAP29_COI ☐ .......... .......... ........A. .......... .......... .......... .......... .......... 639
HAP12_COI ☐ .......... .......... ........A. .......... .......... .......... .......... ...G...... 639
HAP13_COI ☐ .......... .......... ........A. .......... .......... .......... .......... ...G...... 639
100%
Conservation
0%
```

HAP8_COI	☒	TATCAACACC	TA	651
HAP9_COI	☒	....	..	651
HAP22_COI	☒	....	..	651
HAP23_COI	☒	....	..	651
HAP17_COI	☒	....	..	651
HAP16_COI	☒	....	..	651
HAP21_COI	☒	....	..	651
HAP24_COI	☒	....	..	651
HAP15_COI	☒	....	..	651
HAP10_COI	☒	....	..	651
HAP18_COI	☒	....	..	651
HAP25_COI	☒	....	..	651
HAP38_COI	☒	....	..	651
HAP36_COI	☒	....	..	651
HAP37_COI	☒	....	..	651
HAP11_COI	☒	....	..	651
HAP20_COI	☒	....	..	651
HAP26_COI	☒	....	..	651
HAP39_COI	☒	....	..	651
HAP19_COI	☒	....	..	651
HAP27_COI	☒	....	..	651
HAP1_COI	☐	....	..	651
HAP30_COI	☐	....	..	650
HAP14_COI	☐	....	..	651
HAP32_COI	☐	....	..	651
HAP31_COI	☐	....	..	651
HAP3_COI	☐	....	..	651
HAP4_COI	☐	....	..	651
HAP33_COI	☐	....	..	651
HAP34_COI	☐	....	..	651
HAP2_COI	☐	....	..	651
HAP6_COI	☐	....	..	651
HAP28_COI	☐	....	..	651
HAP7_COI	☐	....	..	651
HAP35_COI	☐	....	..	651
HAP5_COI	☐	....	..	651
HAP29_COI	☐	....	..	651
HAP12_COI	☐	....	..	651
HAP13_COI	☐	....	..	651

Conservation

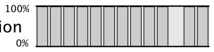

Supplement: S4 File — Marked haplotypes belong to haplogroup A. The non-synonymous mutation highlighted in red is diagnostic for discrimination of A and B haplogroups. (PDF) [file pone.0143297.s004.pdf]
