# Supplementary material for: A COI Nonsynonymous Mutation as Diagnostic Tool for Intraspecific Discrimination in the European Anchovy Engraulis encrasicolus (Linnaeus)
Source: PLoS One. 2015 Nov 24;10(11):e0143297. doi: 10.1371/journal.pone.0143297 (PMC4657973; doi:10.1371/journal.pone.0143297)

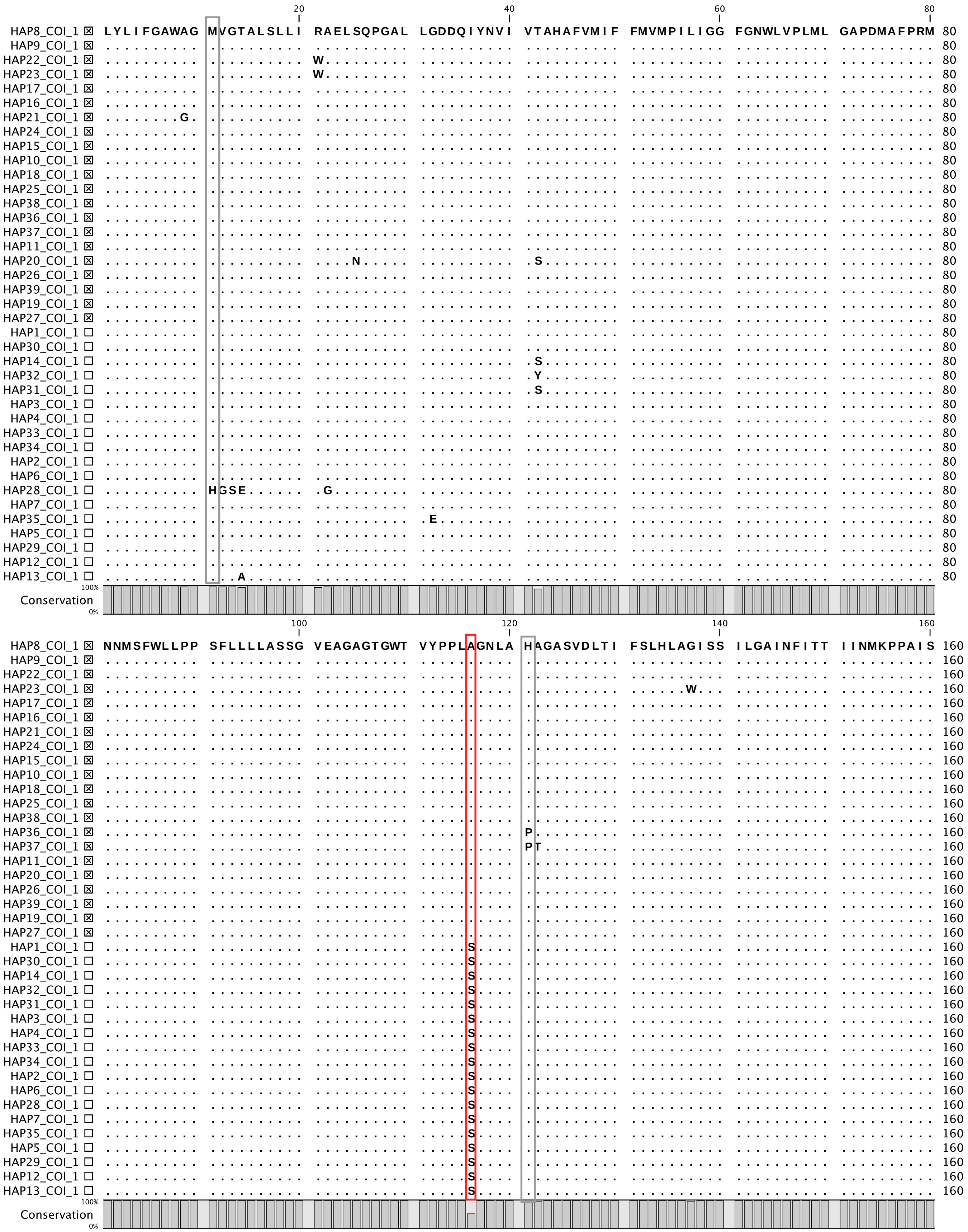
20 40 60 80
HAP8_COI_1 ☒ LYLIFGAWAG MVGTALSLLI RAELSQPGAL LGDDQIYNVI VTAHAFVMIF FMVMPILIGG FGNWLVPLML GAPDMAFPRM 80
HAP9_COI_1 ☒ 80
HAP22_COI_1 ☒ .... W......... 80
HAP23_COI_1 ☒ .... W......... 80
HAP17_COI_1 ☒ 80
HAP16_COI_1 ☒ 80
HAP21_COI_1 ☒ ....G. 80
HAP24_COI_1 ☒ 80
HAP15_COI_1 ☒ 80
HAP10_COI_1 ☒ 80
HAP18_COI_1 ☒ 80
HAP25_COI_1 ☒ 80
HAP38_COI_1 ☒ 80
HAP36_COI_1 ☒ 80
HAP37_COI_1 ☒ 80
HAP11_COI_1 ☒ 80
HAP20_COI_1 ☒ ....N..... .... .S........ 80
HAP26_COI_1 ☒ 80
HAP39_COI_1 ☒ 80
HAP19_COI_1 ☒ 80
HAP27_COI_1 ☒ 80
HAP1_COI_1 ☐ 80
HAP30_COI_1 ☐ 80
HAP14_COI_1 ☐ .... .S........ 80
HAP32_COI_1 ☐ .... .Y........ 80
HAP31_COI_1 ☐ .... .S........ 80
HAP3_COI_1 ☐ 80
HAP4_COI_1 ☐ 80
HAP33_COI_1 ☐ 80
HAP34_COI_1 ☐ 80
HAP2_COI_1 ☐ 80
HAP6_COI_1 ☐ 80
HAP28_COI_1 ☐ .... HGSE...... .G........ 80
HAP7_COI_1 ☐ 80
HAP35_COI_1 ☐ .... .E........ 80
HAP5_COI_1 ☐ 80
HAP29_COI_1 ☐ 80
HAP12_COI_1 ☐ 80
HAP13_COI_1 ☐ ....A...... 80
100%
Conservation
0%
100 120 140 160
HAP8_COI_1 ☒ NNMSFWLLPP SFLLLLASSG VEAGAGTGWT VYPPLAGNLA HAGASVDLTI FSLHLAGISS ILGAINFITT IINMKPPAIS 160
HAP9_COI_1 ☒ 160
HAP22_COI_1 ☒ 160
HAP23_COI_1 ☒ ....W... 160
HAP17_COI_1 ☒ 160
HAP16_COI_1 ☒ 160
HAP21_COI_1 ☒ 160
HAP24_COI_1 ☒ 160
HAP15_COI_1 ☒ 160
HAP10_COI_1 ☒ 160
HAP18_COI_1 ☒ 160
HAP25_COI_1 ☒ 160
HAP38_COI_1 ☒ 160
HAP36_COI_1 ☒ .... P......... 160
HAP37_COI_1 ☒ .... PT........ 160
HAP11_COI_1 ☒ 160
HAP20_COI_1 ☒ 160
HAP26_COI_1 ☒ 160
HAP39_COI_1 ☒ 160
HAP19_COI_1 ☒ 160
HAP27_COI_1 ☒ 160
HAP1_COI_1 ☐ ....S.... 160
HAP30_COI_1 ☐ ....S.... 160
HAP14_COI_1 ☐ ....S.... 160
HAP32_COI_1 ☐ ....S.... 160
HAP31_COI_1 ☐ ....S.... 160
HAP3_COI_1 ☐ ....S.... 160
HAP4_COI_1 ☐ ....S.... 160
HAP33_COI_1 ☐ ....S.... 160
HAP34_COI_1 ☐ ....S.... 160
HAP2_COI_1 ☐ ....S.... 160
HAP6_COI_1 ☐ ....S.... 160
HAP28_COI_1 ☐ ....S.... 160
HAP7_COI_1 ☐ ....S.... 160
HAP35_COI_1 ☐ ....S.... 160
HAP5_COI_1 ☐ ....S.... 160
HAP29_COI_1 ☐ ....S.... 160
HAP12_COI_1 ☐ ....S.... 160
HAP13_COI_1 ☐ ....S.... 160
100%
Conservation
0%

```
                                180                  200
HAP8_COI_1 ☒  QYQTPLFVWA VLITAVLLLL SLPVLAAGIT MLLTDRNLNT TFFDPAGGGD PILYQHL 217
HAP9_COI_1 ☒  .......... .......... .......... .......... .......... ....... 217
HAP22_COI_1 ☒ .......... .......... .......... .......... .......... ....... 217
HAP23_COI_1 ☒ .......... .......... .......... .......... .......... ....... 217
HAP17_COI_1 ☒ .......... .......... .......... .......... .......... ....... 217
HAP16_COI_1 ☒ .......... .......... .......... .......... .......... ....... 217
HAP21_COI_1 ☒ .......... .......... .......... .......... .......... ....... 217
HAP24_COI_1 ☒ .......... .......... .......... .......... .......... ....... 217
HAP15_COI_1 ☒ .......... .......... .......... .......... .......... ....... 217
HAP10_COI_1 ☒ .......... .......... .......... .......... .......... ....... 217
HAP18_COI_1 ☒ .......... .......... .......... .......... .......... ....... 217
HAP25_COI_1 ☒ .......... .......... .......... .......... .......... ....... 217
HAP38_COI_1 ☒ .......... .......... .......... .......... .......... ....... 217
HAP36_COI_1 ☒ .......... .......... .......... .......... .......... ....... 217
HAP37_COI_1 ☒ .......... .......... .......... .......... .......... ....... 217
HAP11_COI_1 ☒ .......... .......... .......... .......... .......... ....... 217
HAP20_COI_1 ☒ .......... .......... .......... .......... .......... ....... 217
HAP26_COI_1 ☒ .......... .......... .......... .......... .......... ....... 217
HAP39_COI_1 ☒ .......... .......... .......... .......... .......... ....... 217
HAP19_COI_1 ☒ .......... .......... .......... .......... .......... ....... 217
HAP27_COI_1 ☒ .......... .......... .......... .......... .......... ....... 217
HAP1_COI_1 ☐  .......... .......... .......... .......... .......... ....... 217
HAP30_COI_1 ☐ .......... .......... .......... .......... ....QQ.EET RFFINTX 217
HAP14_COI_1 ☐ .......... .......... .......... ....V..... ...Y...... ....... 217
HAP32_COI_1 ☐ .......... .......... .......... .......... .......... ....... 217
HAP31_COI_1 ☐ .......... .......... .......... .......... .......... ....... 217
HAP3_COI_1 ☐  .......... .......... .......... .......... .......... ....... 217
HAP4_COI_1 ☐  .......... .......... .......... .......... .......... ....... 217
HAP33_COI_1 ☐ .......... .......... .......... .......... .......... ....... 217
HAP34_COI_1 ☐ .......... .......... .......... .......... .......... ....... 217
HAP2_COI_1 ☐  .......... .......... .......... .......... .......... ....... 217
HAP6_COI_1 ☐  .......... .......... .......... .......... .......... ....... 217
HAP28_COI_1 ☐ .......... .......... .......... .......... .......... ....... 217
HAP7_COI_1 ☐  .......... .......... .......... .......... .......... ....... 217
HAP35_COI_1 ☐ .......... .......... .......... .......... .......... ....... 217
HAP5_COI_1 ☐  .......... .......... .......... .......... .......... ....... 217
HAP29_COI_1 ☐ .......... .......... .......... .......... .......... ....... 217
HAP12_COI_1 ☐ .......... .......... .......... .......... .......... ....... 217
HAP13_COI_1 ☐ .......... .......... .......... .......... .......... ....... 217
```

Conservation

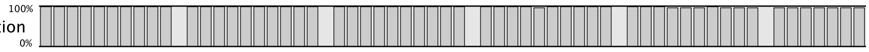

Supplement: S5 File — The codon 116 highlighted in red is diagnostic for discrimination of A and B haplogroups. The codons 11 and 121 highlighted in grey are under episodic positive selection. (PDF) [file pone.0143297.s005.pdf]
